# Molecular thermal vibration assisted 1208-nm excitable photon upconversion with large anti-Stokes shifts

**DOI:** 10.1126/sciadv.aed9451

**Published:** 2026-11-13

**Authors:** Lin-Han Jiang, Ran Li, Ying-Ze Li, Dong-Xue Guo, Yun-Rong Li, Junxue Liu, Ling Huang, Dai-Wen Pang

**Affiliations:** ^1^ Tianjin Key Laboratory of Biosensing and Molecular Recognition, Research Center for Analytical Sciences, Frontiers Science Center for New Organic Matter, Haihe Laboratory of Sustainable Chemical Transformations, College of Chemistry, Nankai University, Tianjin 300071, P. R. China.; ^2^ Time-Tech Spectra, Co., Ltd., Dalian 116085, P. R. China.

## Abstract

The anti-Stokes shift, a critical parameter reflecting the conversion of low-energy photons into high-energy photons, has been constrained by energy losses inherent to multi-step energy transfer processes. Here, we have demonstrated quantum dot–based photon upconversion under 1208-nanometer illumination (below the silicon bandgap, ∼1107 nanometer) and achieved unprecedented anti-Stokes shifts of 1.21 and 1.26 electron volt, which exceed the energy of a single excitation photon (1.03 electron volt). This breakthrough partially arose from the ideal energy cascade between quantum dots and ligands (NKU-0), coupled with nearly energy-lossless triplet fusion of the annihilators. Furthermore, we have found that the ground-state thermal vibrations of tetracene derivatives can promote the population of electrons to high vibrational states, which facilitated the endothermic triplet energy transfer from the ligand to the annihilator, thereby substantially enlarging the anti-Stokes shift. These findings present a feasible design route toward NIR-II–excitable photon upconversion with large anti-Stokes shifts by precisely tailoring quantum dot–based sensitizers and annihilator units, which promotes the efficient exploitation of low-energy photons in chemistry, materials science, and photonics.

## INTRODUCTION

Photon upconversion materials convert low-energy photons into high-energy photons (*1*–*3*), exhibiting enormous potential applications in solar energy harvesting (*4*, *5*), photocatalysis (*6*, *7*), stereoscopic three-dimensional (3D) printing (*8*, *9*), etc. Central to their performance is the anti-Stokes shift, defined as the energy gap between the upconversion maximum emission energy and the excitation energy. To date, there are two main types of continuous light-excitable upconversion materials (Fig. 1) (*1*). One is lanthanide-doped inorganic upconversion nanoparticles (UCNPs), whose emission process relies on the abundant *f*-electron configurations of lanthanide ions (*10*, *11*). In particular, energy transfer upconversion (ETU) is the most popular mechanism, which consists of two components, the sensitizer and activator, such as Yb^3+^/A^3+^ (A = Er, Tm, Ho, Pr) (*3*, *12*). However, the energy mismatches between the sensitizer and the activator, and the nonradiative relaxation process of the activator cause photoexcitation energy loss to reduce the anti-Stokes shift. For instance, in Yb^3+^/Tm^3+^-doped UCNPs, the emission of two-photon upconversion occurs only at 800 nm under 980-nm illumination (Fig. 1A) (*13*). Another type is triplet fusion upconversion (TFU) comprising the photosensitizer and annihilator, which features high upconversion efficiency, low excitation intensity, and tunable excitation/emission wavelengths (Fig. 1B) (*2*, *14*, *15*). In general, the maximum anti-Stokes shift of TFU is the energy of a single excitation photon, i.e., two low-energy photons are converted into one high-energy photon without energy loss. However, the intersystem crossing (ISC) process of the photosensitizer, the triplet energy transfer (TET) from the photosensitizer to the annihilator, and the triplet fusion (TF) process of the annihilator all lead to the photoexcitation energy loss, such that the anti-Stokes shifts of most TFU materials remain lower than the energy of a single excitation photon (*16*, *17*).

**Fig. 1.**
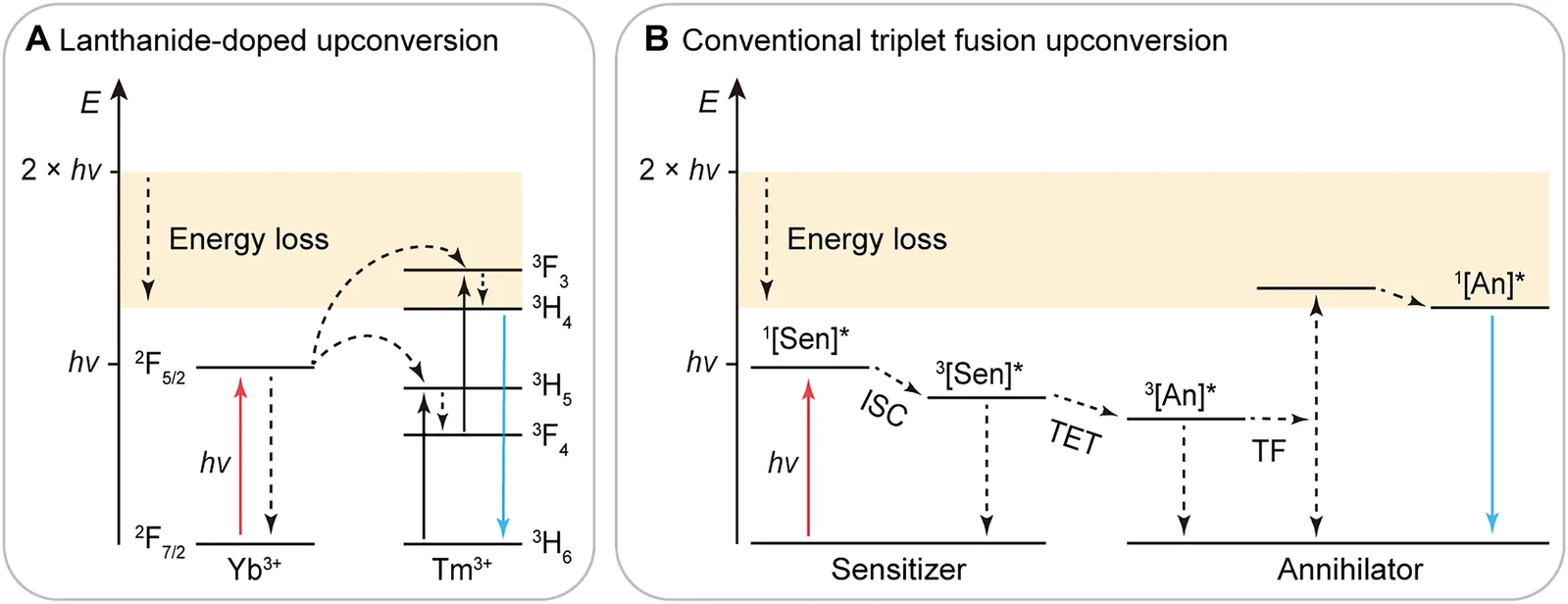
Two main processes for photon upconversion under continuous light excitation. (**A**) Schematic of lanthanide-doped upconversion based on ETU mechanism. (**B**) Diagram of conventional TFU. ISC, TET, and TF denote intersystem crossing, triplet energy transfer, and triplet fusion, respectively. The gap between upconversion emission and twofold photon energy (2 × *hv*) represents the part of the anti-Stokes shift below the energy of a single excitation photon due to energy loss.

According to the photophysical mechanism of TFU, the reduction of energy loss in multistep processes is the only approach to expand the anti-Stokes shift. Quantum dots (QDs), as emerging photosensitizers can avoid the energy loss during ISC because their spin quantum numbers are ill-defined and excited-state energy levels are closely spaced (*18*–*20*). However, QDs generally require surface functionalization with triplet-accepting ligands (*21*, *22*). The substantial energy gap (0.1 to 0.5 eV) between QDs exciton energy and ligand triplet energy is often required to drive Dexter-like energy transfer but imposes unavoidable energy loss (*23*, *24*). We previously mitigated this problem through ligand conformation modulation, yet the anti-Stokes shifts remained lower than the energy of a single excitation photon (*25*).

Here, we rationally designed the surface ligand (NKU-0) and accurately determined its triplet energy level to be 1.0 eV via QD-sensitized phosphorescence measurement. This ligand formed an optimal energy cascade with 1208-nm absorbing lead sulfide QDs (PbS-3.76 QDs), enabling the effective triplet exciton transfer while minimizing energy loss. The annihilators were tetracene derivatives, specifically 5,6,11,12-tetraphenyltetracene (Rubrene) and 5,12-bis((triisopropylsilyl)ethynyl)tetracene (TIPS-Tc). These molecules exhibited twice the triplet energy (2.2 to 2.4 eV) as nearly isoenergetic as the singlet energy (2.2 to 2.3 eV), thus effectively eliminating energy loss during the TF process. The thermal vibration in these annihilators promoted the population of electrons into the high vibrational energy levels of the ground state, forming a “hot band” to facilitate endothermic TET. TFU, in particular, involved two thermally assisted triplet sensitizations to fully use the ambient heat and thus expand the anti-Stokes shift. Under 1208-nm illumination, we directly observed photon upconversion with anti-Stokes shifts as high as 1.21 eV for PbS-3.76/NKU-0/Rubrene and 1.26 eV for PbS-3.76/NKU-0/TIPS-Tc. Both values exceed the energy of a single 1208-nm excitation photon (1.03 eV). These results demonstrate that precise modulation of multistep energy transfer processes achieves photon upconversion with anti-Stokes shifts exceeding the energy of a single excitation photon. This breakthrough provides an effective route for developing high-performance upconversion materials with large anti-Stokes shifts and broadens their applications in low-energy photon harvesting and conversion.

## RESULTS

The versatile chemical tunability of diketopyrrolopyrrole enabled the rational design of surface ligands. Starting from compound 2 (*T*_1_ = 1.1 eV) (*26*, *27*), we synthesized the target ligand NKU-0, in which the extended thiophene moiety reduced the *T*_1_ energy level, and the carboxylic acid group acted as an anchoring site (Fig. 2A). Nuclear magnetic resonance and high-resolution mass spectrometry confirmed successful synthesis of NKU-0 (figs. S1 and S2). The photophysical properties of NKU-0 are summarized in table S1. The comprehensive spectroscopic analysis of NKU-0 revealed the maximum absorption wavelength of 560 nm and emission wavelength of 625 nm, with spectral intersection establishing a singlet energy level of 2.0 eV (Fig. 2B). The absolute fluorescence quantum yield was 22% with a lifetime of 2.2 ns (fig. S3). Compared to the previously reported thiophene-substituted DPP (Th-DPP), NKU-0 exhibits a remarkable reduction in fluorescence quantum yield. This decrease is attributed to two main factors. First, the additional thiophene ring increases molecular rotational freedom, thereby promoting nonradiative decay (*28*). We performed potential energy surface scans of the thiophene ring in Th-DPP and the extended thiophene ring in NKU-0 at the optimized *S*_1_ configuration. The results showed that, in addition to the rotation of the thiophene ring similar in Th-DPP, the extended thiophene ring in NKU-0 increased the rotational freedom and exhibited an even greater tendency to rotate, with rotations occurring within a 40° range at room temperature (fig. S4). A summary of fluorescence quantum yields in thiophene-substituted DPP derivatives shows a decrease with an increasing number of thiophene groups, consistent with the behavior observed in NKU-0 (*29*). Second, the carboxylic group in NKU-0 could further reduce the fluorescence quantum yield. On the one hand, it may enhance interactions with the local environment, leading to an accelerated nonradiative decay rate (*30*). On the other hand, the carboxyl group, acting as an electron-withdrawing substituent directly attached to the DPP-conjugated backbone, enhances the intramolecular charge transfer characteristics of NKU-0, which may further reduce the fluorescence quantum yield (*31*). This was supported by the distinct spatial separation between the HOMO and LUMO on the extended thiophene rings (fig. S5).

**Fig. 2.**
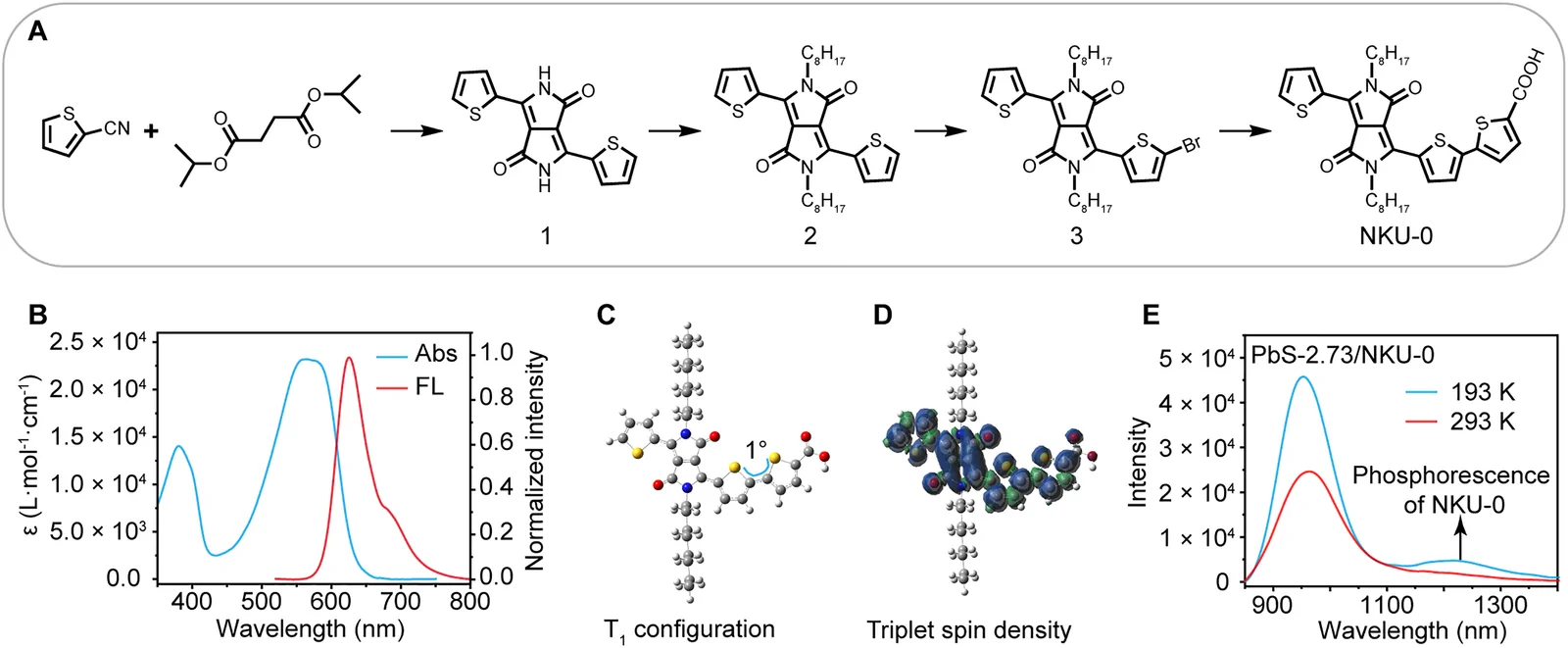
Rational design and properties of NKU-0. (**A**) Synthesis procedure of NKU-0. (**B**) Ultraviolet-visible absorption and emission spectra of NKU-0 in CHCl_3_ at 10 μM. (**C**) Optimized triplet configuration and the dihedral angle between the extended thiophene group and the core. (**D**) Triplet spin density at the optimized triplet configuration. Calculations were performed using ORCA at the B3LYP-D3BJ/def2-TZVP level. (**E**) Photoluminescence spectra of PbS-2.73/NKU-0 at 193 and 293 K. The luminescent component at ∼1215 nm was assigned to the phosphorescence of NKU-0 induced by the heavy-atom effect of the QDs.

To obtain the triplet property of NKU-0, we used theoretical chemical calculations for further study. The dihedral angle between the extended thiophene group and the core was 1° under the optimized triplet configuration, which favored a larger electron delocalization (Fig. 2C). The triplet spin density further indicated a reduced *T*_1_ energy level compared to compound 2 (Fig. 2D). It is worth noting that the spin-forbidden nature of conventional organic small molecules prevents the accurate determination of their *T*_1_ energy levels through phosphorescence emission. However, we found that QD sensitization provided an effective way to solve this problem. We modified NKU-0 onto the PbS-2.73 QDs reported in our previous work by ligand exchange (*25*), in which case NKU-0 was sensitized to the *T*_1_ state very efficiently (fig. S6). Temperature-dependent photoluminescence revealed the emergence of a distinct 1215-nm emission feature in PbS-2.73/NKU-0 at 193 K, absent in control measurements of unfunctionalized PbS-2.73 QDs (Fig. 2E and fig. S7). This luminescent component was assigned to the phosphorescence of NKU-0 induced by the heavy-atom effect of QDs, implying a triplet energy level of 1.0 eV. This also confirmed that NKU-0 as a ligand could form relatively strong electronic coupling with PbS QDs, which would ensure efficient exciton transfer at a small energy gap. We selected 193 K for measurements based on several considerations. The freezing point of toluene is ∼178 K; at 193 K, toluene remains liquid, providing a homogeneous and low-scattering sample environment. Lowering the temperature further causes toluene to solidify into an opaque solid, which substantially increases light scattering and sharply decreases overall photoluminescence intensity. The spectrum at lower temperature (143 K) showed that the photoluminescence intensity decreased substantially, without any noticeable narrowing of the phosphorescence peaks or any improvement in the resolution of the 0-0 transition (fig. S8). Therefore, 193 K offers a suitable balance between signal-to-noise ratio and spectral resolution. Under these conditions, we observed the clear phosphorescence peak and estimated the triplet energy level to be ∼1.0 eV. Considering the broad peak shape, we estimate the *T*_1_ energy level to lie within 0.95 to 1.05 eV (corresponding to wavelengths of 1181 to 1305 nm), which adequately reflects the measurement uncertainty.

We verified the reliability of this approach by performing the same experiments with the previously reported Th-DPP ligand (fig. S9) (*25*). When the temperature was lowered to 193 K, PbS-2.73/Th-DPP also showed a new luminescent component but at ∼1120 nm (fig. S10). This phosphorescence corresponded to the *T*_1_ energy level of 1.1 eV, in agreement with the literature (*26*, *27*). Therefore, we are sure that this method is reliable for the accurate determination of molecular triplet energy levels.

PbS-3.76 QDs were synthesized as light-absorbing antennas based on the reported method (*32*), and the basic photophysical properties were summarized in table S2. The first exciton absorption peak was situated at 1113 nm, allowing the sufficient absorption of photons with energies below the silicon bandgap (∼1107 nm). The maximum photoluminescence wavelength was 1237 nm with a photoluminescence quantum yield of 62%, and the bandgap was determined to be 1.0 eV (*33*). In addition, transmission electron microscopy (TEM) showed a uniform particle size of 3.78 ± 0.19 nm (fig. S11).

Ligand exchange with NKU-0 (600 μM) yielded the photosensitizer (PbS-3.76/NKU-0), whose absorption spectrum showed the characteristic absorption features of both NKU-0 and PbS-3.76 (Fig. 3A). Using the molar extinction coefficient and the absorption spectra of PbS-3.76/NKU-0, we estimated the number of NKU-0 molecules on the surface of PbS-3.76 to be about 51. Functionalization with NKU-0 resulted in 63% photoluminescence quenching and a reduced lifetime under 808-nm excitation, indicating efficient triplet exciton transfer from PbS-3.76 to NKU-0 (Fig. 3, B and C). We also performed photoluminescence quenching experiments under 1208-nm excitation. The results were largely consistent with those obtained under 808-nm excitation, with a quenching efficiency of 58%, demonstrating that triplet exciton transfer behavior is essentially the same under 808- and 1208-nm excitation (fig. S12).

**Fig. 3.**
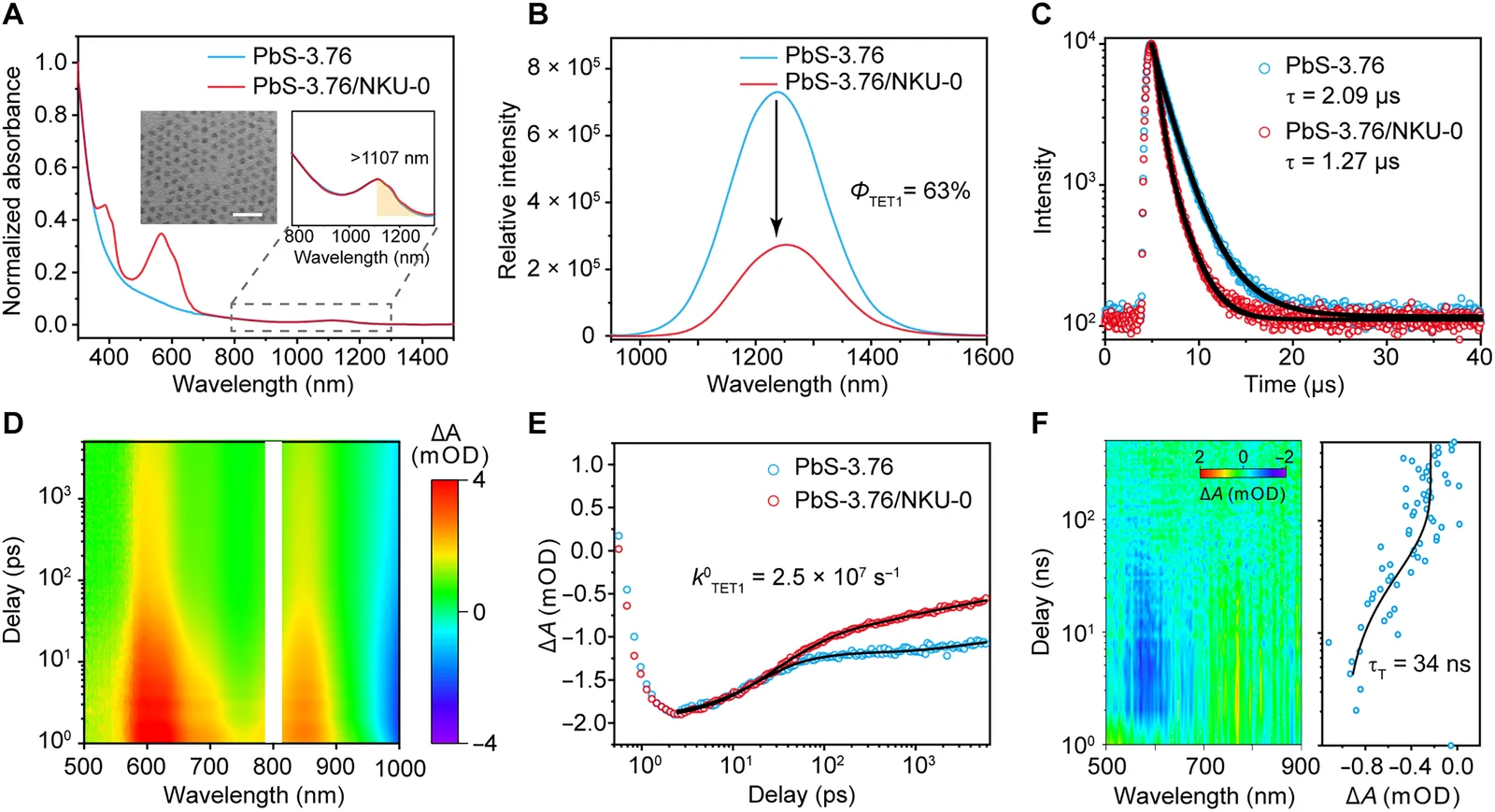
Triplet exciton transfer from PbS-3.76 to NKU-0. (**A**) The absorption spectra of PbS-3.76 and PbS-3.76/NKU-0. The inset shows the TEM image of PbS-3.76 QDs, and the scale bar is 20 nm. (**B**) Relative photoluminescence intensities of PbS-3.76 and PbS-3.76/NKU-0 in argon-saturated toluene, 1 μM, λ_ex_ = 808 nm. Φ_TET1_ represents the triplet exciton transfer efficiency from PbS-3.76 to NKU-0. (**C**) Time-resolved photoluminescence spectra of PbS-3.76 and PbS-3.76/NKU-0 in argon-saturated toluene and corresponding fitting results. Measurements were performed at 1 μM, λ_ex_ = 808 nm. (**D**) Two-dimensional pseudo-color fs-TA spectra of PbS-3.76/NKU-0 following 800-nm excitation. (**E**) Bleach recovery kinetics of PbS-3.76 and PbS-3.76/NKU-0 probed at 990 nm with 800-nm excitation. The intrinsic triplet exciton transfer rate from PbS-3.76 to one NKU-0 is known as *k*^0^_TET1_. (**F**) Double-difference nanosecond transient absorption spectra after subtracting the QDs contribution from PbS-3.76/NKU-0′. On the right is the corresponding kinetics of NKU-0 ground-state bleaching. τ_T_ represents the triplet lifetime of NKU-0. PbS-3.76/NKU-0′ represents the sample with a low NKU-0 loading on the surface of PbS-3.76 QDs, designed to better extract the triplet characteristics of NKU-0.

We used 9-anthracenecarboxylic acid (ACA) as a control to indicate that ligand exchange did not introduce defect states, since no changes were observed in photoluminescence intensity and lifetime after ACA exchange (fig. S13). Thus, we have realized efficient triplet exciton transfer when the QD bandgap was almost isoenergetic with the triplet energy of NKU-0, which was attributed to the strong electronic coupling between the two.

We implemented femtosecond transient absorption (fs-TA) spectroscopy to acquire a better understanding of the triplet exciton transfer process (Fig. 3D and fig. S14). Under selective excitation with an 800-nm femtosecond pulsed laser, the negative signal observed after 950 nm was attributed to the exciton bleaching (XB) of PbS-3.76. The XB recovery of PbS-3.76/NKU-0 was more rapid than that of PbS-3.76, indicating the presence of a triplet exciton transfer process (*18*). Global fitting the XB recovery kinetics at 990 nm yielded the triplet exciton transfer rate (*k*_TET1_) and the intrinsic exciton transfer rate (*k*^0^_TET1_ = *k*_TET1_/*N*_NKU-0_) has been estimated to be 2.5 × 10^7^ s^−1^ (Fig. 3E and table S3). Because of the strong absorption of PbS-3.76/NKU-0 in the visible region, the probe light became saturated and the signal was distorted in the 500- to 600-nm range, resulting in notable signal fluctuations (Fig. 3D). To better obtain the triplet characteristics of NKU-0, we prepared PbS-3.76/NKU-0′ samples with low NKU-0 loading by reducing the concentration of NKU-0 during the ligand exchange process (200 μM) (fig. S15). We then performed femtosecond and nanosecond transient absorption measurements and subtracted the QDs’ contribution through spectral subtraction (Fig. 3F and figs. S16 and S17). In the 500- to 650-nm range, we observed a distinct negative signal corresponding to the absorption of NKU-0, which we assigned to the ground-state bleaching (GSB) of NKU-0. Given that NKU-0 cannot be directly excited by 800-nm light (fig. S18), we attributed this GSB signal to the reduced ground-state population of NKU-0 after sensitization to the triplet state by PbS-3.76 QDs. However, because of the weak excited-state absorption (ESA) of NKU-0 at the triplet state (^3^NKU-0^*^) and its notable overlap with the ESA of the QDs, we were unable to directly observe the ESA signal of the ^3^NKU-0^*^, consistent with our previous studies on similar molecules (*25*). Furthermore, by fitting the GSB kinetics, we found that the triplet lifetime of NKU-0 on the PbS-3.76 surface was ∼34 ns (Fig. 3F). The following factors account for the exceptionally short triplet lifetime. The *T*_1_ energy level of NKU-0 is relatively low (∼1.0 eV). According to the energy gap law, the rate of nonradiative relaxation increases exponentially as the energy gap between the excited state and the ground state decreases (*34*). Furthermore, the heavy-atom effect of the PbS-3.76 QDs reduces the triplet lifetime of NKU-0 (*35*).

PbS-3.76/NKU-0 exhibits intense absorption beyond 1107 nm, providing a foundation for using photons with energies below the silicon bandgap. In conjunction with Rubrene and TIPS-Tc as annihilators, we constructed a 1208-nm excitable upconversion system (Fig. 4A). The spectrum of the excitation source (1208-nm laser) has been shown in fig. S19. To our knowledge, this is the longest wavelength used to excite TFU materials (Fig. 4B and table S4) (*4*). Under 1208-nm irradiation, bright yellow upconversion luminescence was directly observed (Fig. 4C). To account for the high concentration of annihilators and the self-absorption of PbS-3.76/NKU-0 in the visible region, which resulted in the distortion of upconversion emission spectra, we further corrected the upconversion spectra (*25*, *36*). The upconversion intensity underwent a transition from quadratic to linear dependence with respect to the incident power density, revealing the characteristic of the TFU mechanism. Threshold power densities (*I*_th_) of 104 W/cm^2^ for PbS-3.76/NKU-0/Rubrene and 94 W/cm^2^ for PbS-3.76/NKU-0/TIPS-Tc were determined from these crossover points (Fig. 4, D and E). *I*_th_ is closely related to the absorption coefficient at the excitation wavelength, the TET efficiency, the second-order annihilation constant for TF, and the triplet lifetime of the annihilator (*37*, *38*). Because of the short triplet lifetime of NKU-0 and the endothermic transfer, the TET efficiency from NKU-0 to the annihilator is very low, which is the primary reason for the higher *I*_th_. The microsecond upconversion luminescence lifetimes provided additional evidence in favor of the TFU mechanism (fig. S20).

**Fig. 4.**
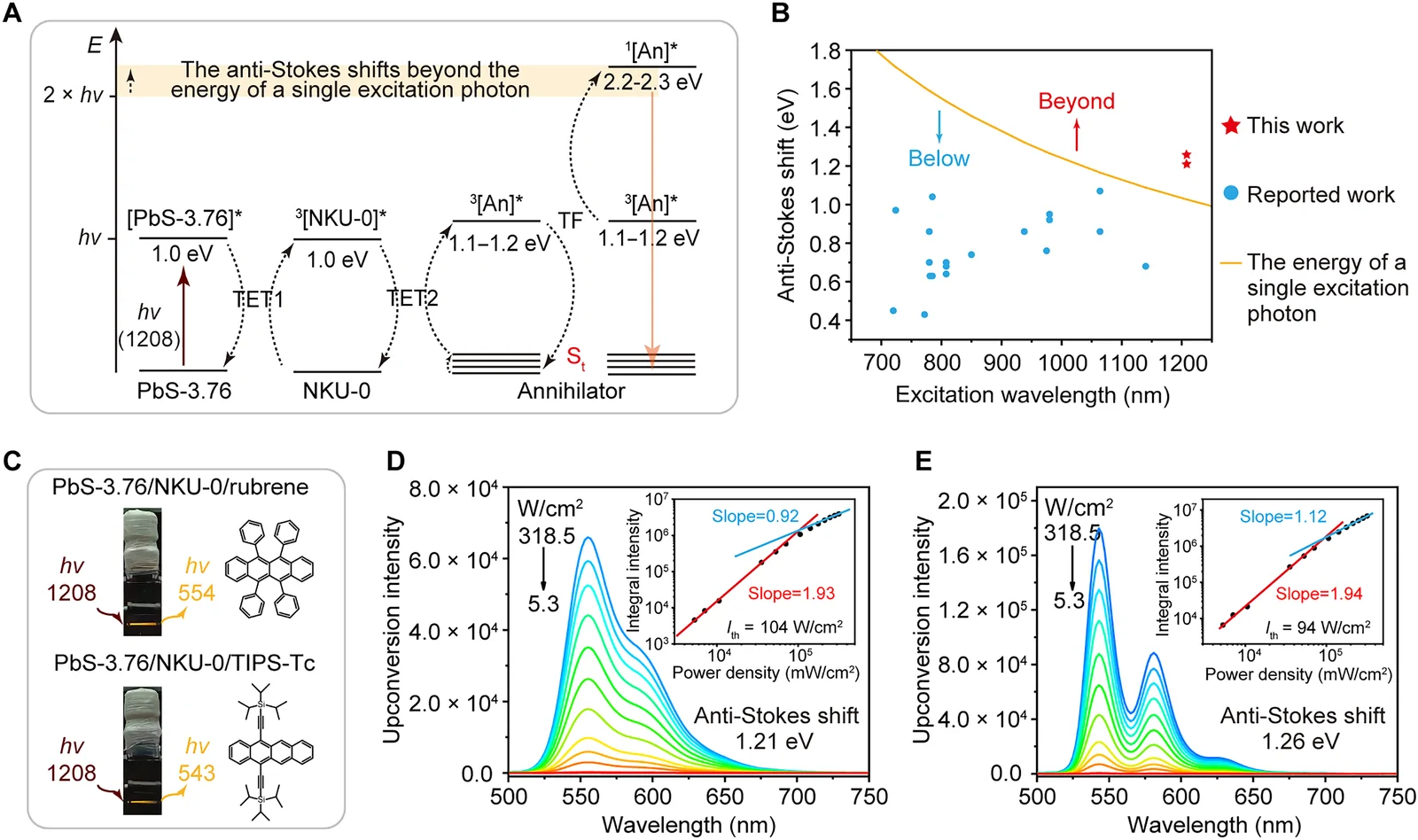
Photon upconversion with anti-Stokes shifts exceeding the energy of a single excitation photon. (**A**) Schematic of TFU with anti-Stokes shifts exceeding the energy of a single excitation photon under 1208 nm continuous light excitation. *S*_t_ represents a higher vibrational energy level of the ground state. The gap between upconversion emission and twofold photon energy (2 × *hv*) is the part of the anti-Stokes shift beyond the energy of a single excitation photon. (**B**) Current status of studies on near-infrared photoexcited TFU. (**C**) Photographs under 1208 nm laser irradiation in airtight cuvettes. The yellow emission comes from upconversion. On the right are the annihilator structures. Power-dependent TFU spectra of (**D**) PbS-3.76/NKU-0/Rubrene and (**E**) PbS-3.76/NKU-0/TIPS-Tc with various power densities (5.3 to 318.5 W/cm^2^). Insets: dependence of the TFU intensity on the incident power density.

Notably, despite the short triplet lifetime of NKU-0 on the surface of PbS-3.76 QDs, NKU-0 is still able to overcome the triplet exciton transfer barrier imposed by the oleic acid layer. No upconversion emission was observed with unfunctionalized PbS/Rubrene and PbS/TIPS-Tc (fig. S21), which further supports this finding. We recorded anti-Stokes shifts of 1.21 eV for PbS-3.76/NKU-0/Rubrene and 1.26 eV for PbS-3.76/NKU-0/TIPS-Tc, exceeding the energy of a single excitation photon of 1.03 eV for 1208-nm excitable TFU materials (Fig. 4B and table S4).

## DISCUSSION

Typically, multistep energy transfer leads to photoexcitation energy loss, and thus the anti-Stokes shifts of previously reported upconversion materials remain well below the energy of a single excitation photon. Our results confirm that anti-Stokes shifts exceeding the energy of a single excitation photon can be achieved in triplet-fusion upconversion via rational design. First, QDs as photosensitizers can eliminate significant ISC loss. Second, the easily modifiable nature of diketopyrrolopyrrole allowed us to synthesize the ligand on demand. In particular, we found that NKU-0 could be sensitized by PbS-2.73 to produce phosphorescence, which overcame the spin-forbidden character owing to the heavy-atom effect of the QDs. On the one hand, this could be used to accurately determine the triplet energy levels of organic chromophores. On the other hand, it confirmed the relatively strong electronic coupling between NKU-0 and QDs, which accounted for the efficient triplet exciton transfer from PbS-3.76 in the absence of an energy-gap driving force (*25*, *39*, *40*).

In addition, the combined energy of two triplet states (2.2 to 2.4 eV) is close to that of the singlet state (2.2 to 2.3 eV) for tetracene derivatives (*41*, *42*), which minimized the energy loss during the TF process. However, achieving anti-Stokes shifts exceeding the energy of a single excitation photon requires not only minimal energy loss across all TFU processes but also supplementary energy such as ambient heat. It has been reported that some fluorescent molecules—such as oxazine (*43*), porphyrin (*44*, *45*), and rhodamine (*46*)—can absorb low-energy photons to produce anti-Stokes–shifted luminescence (*47*). We found that the tetracene derivatives also showed this characteristic. When we used low-energy photons (660 nm) to excite Rubrene and TIPS-Tc, high-energy fluorescence emission was observed (Fig. 5, A and B). Therefore, by absorbing thermal energy from the surroundings, electrons can be promoted to higher vibrational energy levels of the ground state, which contributes to the endothermic TET from the ligand to the annihilator. The triplet-fusion strategy involves two endothermic triplet sensitization steps, thereby doubling the energy gain.

**Fig. 5.**
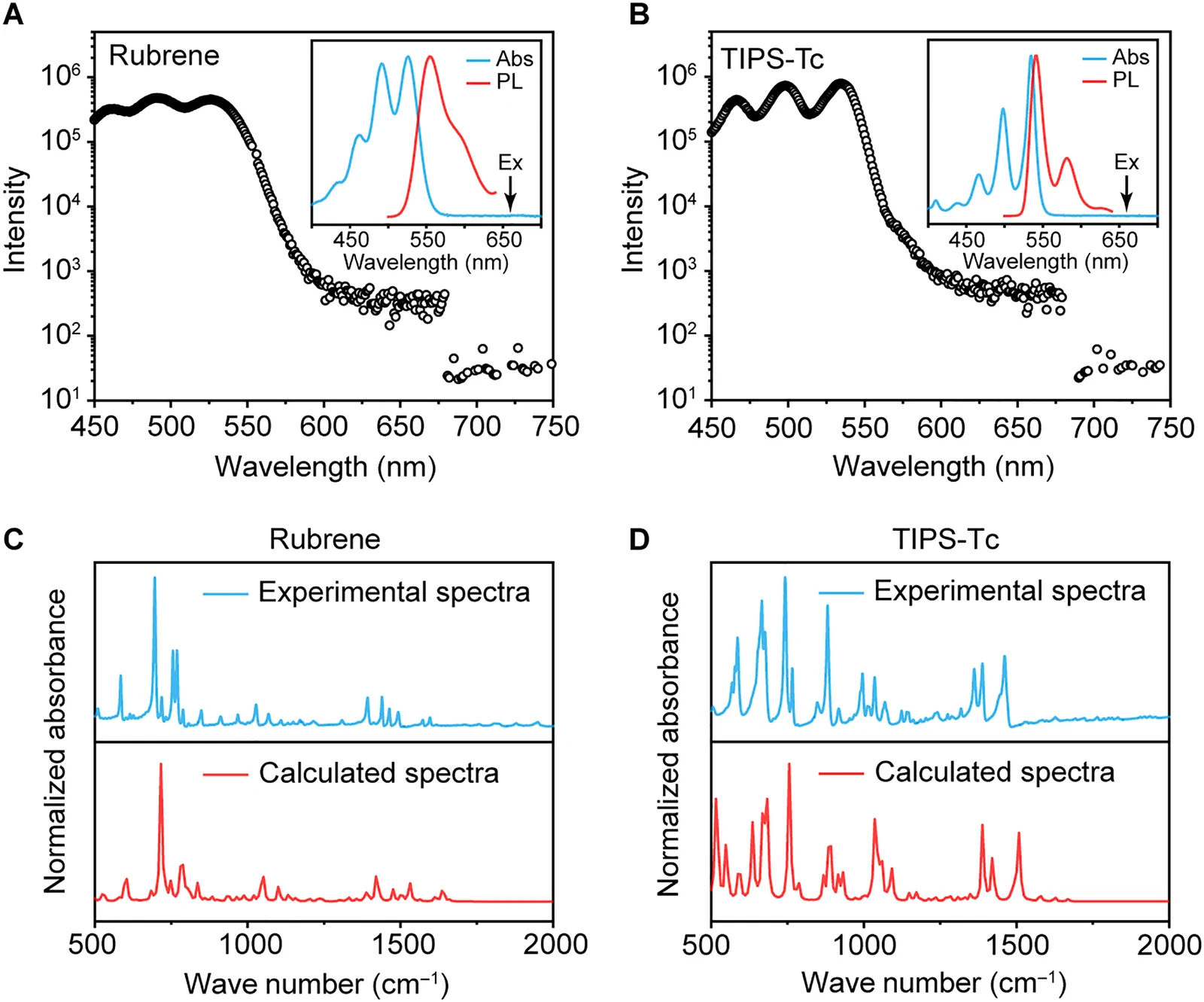
The high vibrational energy levels of the ground state of the annihilator. Excitation spectra of (**A**) Rubrene and (**B**) TIPS-Tc. The insets show absorption and emission spectra under 660-nm excitation of the corresponding molecules. Experimental and calculated infrared spectra of (**C**) Rubrene and (**D**) TIPS-Tc. Calculations were performed with Gaussian 09 at the B3LYP/def2-TZVP level in toluene.

We performed a quantitative analysis using the Arrhenius equation (*48*, *49*). The activation energy was approximated as the energy difference associated with the TET process from NKU-0 (*T*_1_ = 1.0 eV) to the annihilator triplet state (*T*_1_ = 1.1 to 1.2 eV). At room temperature, for an endothermic TET with an energy gap of 0.1 to 0.2 eV, the transfer rate is 50 to 2000 times slower than that of an isoenergetic TET. The thermally assisted vibrational mechanism uses ambient thermal energy to populate high vibrational levels of the annihilator, thereby effectively reducing the energy barrier and accelerating the TET from the NKU-0 ligand to the annihilators. We calculated the infrared spectra of the two molecules, which were in agreement with the experimental spectra (Fig. 5, C and D). The absorption between 800 and 1600 cm^−1^ corresponds to vibrational modes with activation energies ∼0.1 to 0.2 eV.

To gain a deeper understanding, we conducted temperature-dependent upconversion measurements of PbS-3.76/NKU-0/Rubrene and PbS-3.76/NKU-0/TIPS-Tc. As shown in fig. S22, the upconversion intensity increased substantially with temperature (from 233 to 333 K). Raising the temperature may introduce two contributing factors to improve upconversion intensity. One is the molecular thermal-vibration effect. Elevated temperature increases the population of high-vibrational ground-state levels of the annihilator and lowers the energy barrier for endothermic TET from the ligand to the annihilator. The other is the intermolecular diffusion-collision effect. Higher temperature enhances molecular diffusion and collision frequency and raises the effective collision probability between triplet-state ligands and annihilators. The former directly reduces the energy barrier for endothermic TET, whereas the latter improves intermolecular collision efficiency. These two effects promote endothermic TET from intramolecular and intermolecular perspectives, respectively, and together boost TFU performance under 1208-nm excitation. However, these two effects are difficult to separate fully in the current experiments. In addition, we measured the temperature-dependent fluorescence spectra of Rubrene and TIPS-Tc. The results reveal an increase in fluorescence intensity as the temperature rose from 233 to 333 K, confirming the thermally vibrational behavior of the tetracene derivatives in the singlet state (fig. S23). The anti-Stokes shift of the upconversion emission depends on the fluorescence emission of the annihilator and the photoexcitation wavelength (*50*). Since the fluorescence spectrum of the annihilator shows no notable shift with temperature, the anti-Stokes shift of TFU remains unchanged across the temperature range. By combining temperature-dependent measurements, excitation spectra, and infrared spectra, we confirmed that molecular thermal vibrations of the annihilator facilitate endothermic TET from NKU-0, thus enabling anti-Stokes shifts that exceed the energy of a single excitation photon.

We also conducted a series of control experiments to exclude alternative mechanisms. Under the same conditions, no upconversion emission was detected using PbS-3.76/ACA as the photosensitizer compared to the PbS-3.76/NKU-0 (fig. S24). The same results were observed in the system using PbS-3.76 as the photosensitizer without ligand exchange (fig. S21). This indicates that upconversion with anti-Stokes shifts exceeding the energy of a single excitation photon depends on the presence of the NKU-0 surface ligand. Furthermore, the continuous-wave laser used was insufficient to induce multiphoton absorption in PbS-3.76 QDs, as confirmed by the linear relationship between the photoluminescence intensity of the QDs and excitation intensity (fig. S25). In addition, the upconversion efficiencies of PbS-3.76/NKU-0/Rubrene and PbS-3.76/NKU-0/TIPS-Tc showed no wavelength dependence within 808 to 1208 nm (fig. S26), ruling out the influence of hot-exciton non-equilibrium photophysical effects. In practice, once the excitation wavelength and the annihilator are fixed, the anti-Stokes shift is predetermined, and upconversion emission can occur as long as the multistep photophysical processes are operative. Among these, the most challenging step is the endothermic TET from the NKU-0 ligand to the annihilator. The thermally vibration-assisted mechanism uses ambient thermal energy to populate high vibrational levels of the annihilator, effectively reducing the energy barrier and accelerating TET. Consequently, despite the extremely short triplet lifetime of the ligand, endothermic TET can still take place, ultimately yielding upconversion emission with anti-Stokes shifts that surpass the energy of a single excitation photon.

It should be acknowledged that this endothermic TET efficiency is not high enough, thus limiting the upconversion efficiencies (η_UC_, normalized to 100%) to 0.0015% for PbS-3.76/NKU-0/Rubrene and 0.0026% for PbS-3.76/NKU-0/TIPS-Tc. The η_UC_ values remain relatively low compared with existing NIR-excited QD-based TFU. We further analyzed the TFU efficiency using the expression η_UC_ = 2 × Φ_UC_ = 2 × Φ_ex_ × Φ_TET1_ × Φ_TET2_ × Φ_TF_ × Φ_f_ (*22*, *51*). Here, Φ_UC_ represents TFU quantum yield. Because of the two-to-one photon nature of TFU, the Φ_UC_ is normalized to η_UC_ using a coefficient of 2. Φ_ex_ represents the quantum yield of triplet exciton generation in the QDs. Because of the mixed singlet-triplet character in PbS QDs, Φ_ex_ is generally high (typically close to unity) and therefore does not limit the upconversion performance (*40*). Φ_TET1_ is the quantum yield of triplet exciton transfer from the photoexcited QD to the surface ligand, while Φ_TET2_ is the quantum yield of TET from the triplet ligand to the annihilator. Φ_TF_ denotes the TF quantum yield, and Φ_f_ is the fluorescence quantum yield of the annihilator. During the sensitization of NKU-0 by PbS-3.76 QDs to form^3^ NKU-0^*^, we observed a pronounced decrease in both the photoluminescence lifetime and intensity of PbS-3.76 QDs following NKU-0 functionalization (Fig. 3, B and C). This indicated efficient triplet exciton transfer from PbS-3.76 QDs to NKU-0. However, the Φ_TET2_, in which the ^3^NKU-0^*^ further sensitizes the annihilator (Rubrene or TIPS-Tc), is the primary factor responsible for the modest upconversion performance. The Φ_TET2_ is governed by the competition between the intrinsic decay of the ^3^NKU-0^*^ and TET to the annihilator (*40*). The extremely short triplet lifetime of NKU-0 (34 ns) and the endothermic transfer pathway result in an extremely low Φ_TET2_. The thermally vibration-assisted mechanism we propose centers on the sensitization of the annihilator in high vibrational levels by ^3^NKU-0^*^, thereby lowering the effective energy barrier. According to the Boltzmann distribution, ∼2% of the annihilator molecules occupy vibrational states of 0.1 eV at room temperature. Although this fraction is small, meaning that only a small proportion of annihilators can participate in this efficient pathway, we increase the collision probability by using a high annihilator concentration of 20 mM. Meanwhile, because the solution remains in thermal equilibrium, depleted high-vibrational-level annihilator molecules are continuously replenished. Therefore, the short triplet lifetime of NKU-0 is likely the more critical limiting factor.

Moving forward, η_UC_ can be improved by extending the triplet lifetime of surface ligands. For instance, coating PbS QDs with a CdS shell could balance Φ_TET1_ and the triplet lifetime of surface ligands (*52*). Alternatively, ligands with dual strong-weak binding sites could be designed to avoid direct conjugation to the surface of PbS QDs. This would preserve efficient triplet exciton transfer while reducing heavy atom–mediated triplet lifetime quenching (*25*). These strategies are expected to prolong the triplet lifetime of surface ligands, enhance Φ_TET2_, and ultimately improve overall upconversion performance. Although the η_UC_ reported in this study is modest, its core scientific contribution lies in the first experimental demonstration of anti-Stokes shifts exceeding the energy of a single excitation photon under 1208-nm excitation, the longest excitation wavelength reported to date.

The pursuit of large anti-Stokes shifts represents a fundamental challenge in photon upconversion research, as it constitutes a key advantage of upconversion technology. QDs as photosensitizers are promising in the development of TFU with large anti-Stokes shifts because of the negligible ISC energy loss compared with conventional organic photosensitizers. Customized ligand synthesis and strong electronic coupling between QDs and ligands enable efficient triplet exciton transfer with minimal energy loss, which is crucial for the development of QDs/ligand hybrid materials. Moreover, we found that QDs can sensitize molecules to produce phosphorescence, thus providing an effective method to accurately determine molecular triplet energy levels. Tetracene derivatives as annihilators can minimize the energy loss in TF. Furthermore, their electrons can be promoted to higher vibrational energy levels of the ground state by absorbing thermal energy, which promotes endothermic TET. The triplet-fusion mechanism involves two endothermic triplet sensitization steps, thereby doubling the energy gain. Thus, by designing the QD/ligand/annihilator combination, we have realized 1208-nm excited TFU with anti-Stokes shifts exceeding the energy of a single excitation photon (1.03 eV) by ∼0.2 eV. This research promotes the capture and utilization of photons with energies below the silicon bandgap and provides useful insight and clear guidance for achieving large anti-Stokes shifts in upconversion.

## MATERIALS AND METHODS

### Chemicals

All the mentioned chemicals were used as received without further purification unless otherwise noted. Sodium, tetrahydrofuran, oleic acid, oleylamine, 1-octadecene (ODE), and bis(trimethylsilyl)sulfide (TMS_2_S) were purchased from Shanghai Aladdin Biochemical Technology Co. Ltd. 2-Methyl-2-butanol was purchased from Adamas-Beta. Anhydrous ferric chloride was purchased from RHAWN. 2-cyanothiophene, potassium iodide, *N*-bromosuccinimide, tetrakis(triphenylphosphine)palladium [Pd(PPh_3_)_4_], and 2-carboxythiophene-5-boronic acid were purchased from Shanghai Bide Pharma Co. Ltd. 18-crown-6 ether was purchased from Shanghai Jiuding Chemical Technology Co. Potassium carbonate (K_2_CO_3_) was purchased from BENCHMARK. 1-bromooctane, 5,6,11,12-tetraphenyltetracene (Rubrene), and 5,12-bis((triisopropylsilyl)ethynyl)tetracene (TIPS-Tc) were purchased from MERYER. *N*,*N′*-dimethylformamide was purchased from J&K Scientific. Diisopropyl succinate and acetic acid were purchased from TCI. Deuterated dimethyl sulfoxide-d_6_ (DMSO-*d*_6_) was purchased from Innochem. Lead oxide (PbO) was purchased from Alfa Aesar. Toluene was purchased from Tianjin Bohua Chemical Reagent. Methanol (MeOH), ethanol (EtOH), dichloromethane (DCM), hexane, and silica gel (40 to 63 μm) were purchased from the Tianjin Hedong District Guangda Service Department.

### Triplet exciton transfer efficiency from PbS-3.76 to NKU-0

The triplet exciton transfer efficiency from PbS-3.76 to NKU-0 (Φ_TET1_) was calculated using Eq. 1 (*53*). *I* and *I*_0_ represent the photoluminescence intensities of PbS-3.76/NKU-0 and unfunctionalized PbS-3.76, respectively.

$$\Phi_{\text{TET}1}=1-\frac{I}{I_{0}}$$ (1)

The use of the photoluminescence intensity quenching and lifetime quenching to estimate the triplet exciton transfer efficiency relies on the following assumptions.

Under steady-state conditions, the exciton generation rate equals the decay rate

$$G=k_{\text{QDs}}\times N_{0}$$

where *G* is the exciton generation rate, *k*_QDs_ is the intrinsic decay rate of the QDs, and *N*_0_ is the steady-state exciton population. Under these conditions, the photoluminescence intensity is given by

$$I_{0}=k_{r}\times N_{0}=k_{r}\times G\times {k_{\text{QDs}}}^{-1}=k_{r}\times G\times \tau_{0}$$

where *I*_0_ represents the photoluminescence intensity, *k*_r_ represents the radiative decay rate of the QDs, and τ_0_ represents the photoluminescence lifetime of the QDs.

When photoluminescence quenching of QDs results solely from additional triplet exciton transfer, as verified by control experiments with ACA in which no quenching was observed, the exciton generation rate still equals the decay rate

$$G=\left(k_{\text{QDs}}+k_{\text{TET1}}\right)\times N$$

Under the same conditions, the exciton generation rate (*G*) remains constant, while the steady-state exciton population (*N*) changes. The exciton decay rate comprises both the intrinsic decay rate of the QDs (*k*_QDs_) and TET rate from QDs to ligands (*k*_TET1_). Under these conditions, the photoluminescence intensity is given by

$$I=k_{r}\times N=k_{r}\times G\times {\left(k_{\text{QDs}}+k_{\text{TET1}}\right)}^{-1}=k_{r}\times G\times \tau$$

where *I* and τ are the photoluminescence intensity and lifetime of the QDs in the presence of TET to ligands.

Thus, *I*/*I*_0_ = τ/*τ*_0_, meaning that the photoluminescence intensity quenching and lifetime quenching are theoretically equivalent for calculating the triplet exciton transfer efficiency.

In our measurements, the photoluminescence lifetime of PbS-3.76 QDs was notably shortened after NKU-0 ligand binding, approaching the limit of the instrument response function. This introduced some error in the photoluminescence lifetime values obtained via time-resolved photoluminescence, leading to slight discrepancies between the triplet exciton transfer efficiencies calculated from photoluminescence lifetime and intensity. Consequently, we relied on the photoluminescence intensity-based method to calculate the triplet exciton transfer efficiency while using TRPL results qualitatively to confirm trends.

### Transient absorption measurement

The fs-TA of PbS-3.76 and PbS-3.76/NKU-0 was performed using a commercial pump-probe setup (TA-100, Time-Tech Spectra Co. Ltd. Dalian, P. R. China), which was based on a PHAROS laser system from Light Conversion (1030 nm, <290 fs, 200 μJ per pulse, and 100-kHz repetition rate), nonlinear frequency mixing techniques, and the Femto-TA100 spectrometer (Time-Tech Spectra). Briefly, the 1030-nm output pulse from the regenerative amplifier was split in two parts with an 80% beam splitter. The reflected part was used to pump an ORPHEUS optical parametric amplifier (OPA), which generates an 800-nm laser pulse. The transmitted 1030-nm beam was split again into two parts. One part, containing less than 50% of the incident power, was attenuated with a neutral density filter and focused into a 3-mm-thick sapphire window to generate a white-light continuum used for probe beam. The probe beam was focused onto the sample using an Ag parabolic reflector onto the sample. After the sample, the probe beam was collimated and then focused into a fiber-coupled spectrometer with complementary metal-oxide semiconductor sensors. The intensity of the pump pulse used in the experiment was controlled by a variable neutral-density filter wheel. The delay between the pump and probe pulses was controlled by a motorized delay stage. The kinetics of XB recovery at 990 nm were globally fitted to extract the triplet exciton transfer rate (*k*_TET1_).

To better obtain the triplet characteristics of NKU-0, we performed fs-TA of PbS-3.76/NKU-0′ on a Femto-TA100 spectrometer and nanosecond transient absorption of PbS-3.76 and PbS-3.76/NKU-0′ on a Nano-TA100 spectrometer under 800-nm excitation. The contribution of the QDs was then removed by spectral subtractions. Specifically, the fs-TA spectrum of PbS-3.76/NKU-0′ at 20 ps was taken as the characteristic spectrum of the QDs. The contribution of the QDs to the PbS-3.76/NKU-0′ spectra was derived from the ratio of the average intensity of the characteristic QD spectrum in the 830- to 870-nm range to the corresponding average intensity of PbS-3.76/NKU-0′ at different delay times. The double-difference spectra were obtained by subtracting the two. For ns-TA spectra, the spectrum of PbS-3.76 at 5.4 ns was used as the characteristic spectrum of the QDs. All other procedures were performed identically to obtain the double-difference spectra. The kinetics of GSB at 580 nm were fitted to extract the triplet lifetime of NKU-0.

### Photon upconversion measurement

In a glovebox, a mixture of PbS-3.76/NKU-0 and annihilator was sealed in an airtight cuvette with final concentrations of 10 μM for PbS-3.76/NKU-0 and 20 mM for annihilators, in toluene. A 1208-nm continuous-wave laser (MDL-H-1208-1.5 W) was chosen to excite the sample. To account for the high concentration of annihilator and the self-absorption of PbS-3.76/NKU-0 in the visible region, which resulted in the distortion of the upconversion emission, we further corrected the upconversion spectra based on the fluorescence spectra of the annihilator in dilute solution (*25*, *36*).

The η_uc_ was calculated by comparison with the standard [diiodinated BODIPY in acetonitrile, Φ_std_ = 2.7% (*54*)] using Eq. 3 (*35*)

$$\eta_{\text{uc}}=2\times \Phi_{\text{ref}}\times \left(\frac{A_{\text{std}}}{A_{\text{sam}}}\right)\times \left(\frac{I_{\text{sam}}}{I_{\text{std}}}\right)\times {\left(\frac{n_{\text{sam}}}{n_{\text{std}}}\right)}^{2}$$ (2)

A=Laser powerhcλ(1−10−Abs) (3)

The *A*_sam_/*A*_std_ represent the numbers of photons absorbed by the sample/standard, respectively. The *I*_sam_ is the integrated intensity of corrected TFU spectra of the sample, and the *I*_std_ represents the integrated luminescence intensity of the standard. *n*_sam_/*n*_std_ are the solvent refractive indices of the sample/standard, respectively. The upconversion samples were excited at 1208 nm, and the standard was excited at 532 nm.

Lasers with different wavelengths (808, 980, 1064, and 1208 nm) were used to excite the upconversion samples, and relative efficiencies were calculated using the 1208-nm excitation as the reference. Temperature-dependent upconversion spectra were measured using a temperature-controlled stage under 1208-nm excitation.

## Supplementary Materials

### This PDF file includes:

Supplementary Text Figs. S1 to S26 Tables S1 to S4 References
