# Supplementary material for: Molecular thermal vibration assisted 1208-nm excitable photon upconversion with large anti-Stokes shifts
Source: Sci Adv. 2026 Nov 13;12(46):eaed9451. doi: 10.1126/sciadv.aed9451 (PMC13763850; doi:10.1126/sciadv.aed9451)
Supplement: Supplementary file 1 [file sciadv.aed9451_sm.pdf]

Science Advances
AAAS

Supplementary Materials for

Molecular thermal vibration assisted 1208-nm excitable photon upconversion with large anti-Stokes shifts

Lin-Han Jiang *et al.*

Corresponding author: Ling Huang, huangl1@nankai.edu.cn; Dai-Wen Pang, dwpang@whu.edu.cn

Sci. Adv. **12**, eaed9451 (2026)
DOI: 10.1126/sciadv.aed9451

This PDF file includes:

Supplementary Text
Figs. S1 to S26
Tables S1 to S4
References

1. Supplementary Text

1.1 Synthetic procedure of NKU-0

Compound 1 was prepared according to the literature (*25*). Compound 1 (900 mg, 3 mmol), anhydrous K_2CO_3 (2.5 g, 18 mmol), a catalytic amount of KI and 18-crown-6 were added to a 100 mL flask containing 30 ml of argon-saturated anhydrous DMF. When the temperature was heated to 100 °C, 1-bromooctane (3.9 g, 20 mmol in 10 mL of anhydrous DMF) was added. Then, the reaction was carried out at 120 °C for 10 hours. The reaction was cooled to room temperature, extracted and dried with anhydrous Na_2SO_4. The residue was purified by silica gel column chromatography with CH_2Cl_2 to give compound 2.

Compound 2 (262 mg, 0.5 mmol) was added to a 50 mL flask containing 10 mL DCM. Then, NBS (89 mg, 0.5 mmol in 10 mL DCM) was slowly dripped into the reaction system. The reaction was stirred for 6 hours at room temperature away from light. After being extracted and dried with anhydrous Na_2SO_4, the residue was purified by silica gel column chromatography with CH_2Cl_2 : hexane = 1:3 *v/v* to give Compound 3.

Compound 3 (130 mg, 0.2 mmol), 2-carboxythiophene-5-boronic acid (172 mg, 1 mmol), anhydrous K_2CO_3 (138 mg, 1 mmol), and a catalytic amount of $Pd(PPh_3)_4$ were added to a schlenk flask. In a glove box, the mixture of 25 mL anhydrous and argon saturated toluene, ethanol and water (2:2:1) was added. After sealing, the reaction was carried out at 100 °C for 12 hours. Then, the solution was adjusted to pH=5 with 1 M HCl and then extracted and dried with anhydrous Na_2SO_4. The residue was purified by silica gel column chromatography with CH_2Cl_2 : MeOH = 15:1 *v/v* (containing a few drops of acetic acid) to give NKU-0. ^{1}H NMR (400 MHz, DMSO-d_6) δ (ppm): 8.83 (d, J = 3.9 Hz, 1H), 8.80 (d, J = 4.3 Hz, 1H), 8.11 (d, J = 5.0 Hz, 1H), 7.74 (d, J = 4.3 Hz, 1H), 7.63-7.58 (m, 1H), 7.54 (d, J = 3.5 Hz, 1H), 7.41 (t, J = 4.3 Hz, 1H), 4.01 (t, J = 7.2 Hz, 4H), 1.69-1.59 (m, 4H), 1.28-1.19 (m, 20H), 0.86-0.81 (m, 6H). HRMS m/z: $[C_{35}H_{42}N_2O_4S_3\text{-}H]^-$, calculated: 649.2234; found: 649.2236.

1.2 Synthetic procedure of PbS-3.76 QDs

PbS QDs were synthesized according to the reported literature (*32*). A mixture of PbO (0.45 g), oleic acid (2 mL), and ODE (18 mL) was heated to 110 °C in a 50 mL three-neck flask for an hour. Then the reaction temperature was heated and stabilized at 165 °C, and sulfur precursor (0.21 mL TMS_2S in 10 mL anhydrous ODE) was swiftly injected. The heater removed right after injection. 2 min after injection, the reaction flask was rapidly cooled down to room temperature followed by the injection of 10 mL cold hexane. The synthesized QDs were washed three times by adding them to

hexane/ethanol mixture (1:3) and then centrifuged at 8000 rpm for 5 minutes. The final pellet was transferred to glove box and redispersed in toluene for future use. The size of PbS-3.76 was determined by measuring the position of the first exciton peak and verified by transmission electron microscopy, from which the molar extinction coefficient was calculated and used to calculate QDs concentration (*55*). The bandgap of QDs was determined using the Tauc Plot method (*33*).

1.3 Procedure of ligand exchange

In order to exchange oleic acid on the surface of PbS-3.76 QDs by NKU-0 ligand, 10 μM PbS-3.76 was mixed with 600 μM NKU-0 in argon-saturated toluene and stirred for 40 minutes. To better obtain the triplet characteristics of NKU-0, we reduced the concentration of NKU-0 to 200 μM and prepared PbS-3.76/NKU-0' samples with low NKU-0 loading. The synthesized PbS-3.76/NKU-0 were purified by adding 1:5 v/v of toluene/acetone mixture and then centrifuged at 8000 rpm for 10 minutes. The final pellet was quickly transferred to a glovebox and redispersed in toluene for future use.

1.4 Characterization of NKU-0

^{1}H NMR spectra was recorded on a Bruker AVANCE III 400 MHz spectrometer. High-resolution mass spectrometry (HRMS) was performed on a Varian 7.0 T FTMS. The absorption spectra was recorded by a Shimadzu UV-3600 plus spectrophotometer. All photoluminescence (PL) properties were measured on an Edinburgh FLS1000 spectrometer. PL spectra was excited with xenon lamp (500 nm) and the integrating sphere (SM4 from Edinburgh Instruments Ltd.) was used for absolute PL quantum yield. The fluorescence lifetime was excited with a picosecond pulsed laser (405 nm). The luminescence signal was all collected by a visible PMT detector. Measurements were performed in $CHCl_3$ at 10 μM. The optimized triplet configuration and triplet spin density were calculated on Orca at B3LYP/G-D3BJ def2-TZVP level. The potential energy surface scans were performed on Gaussian 09 at B3LYP/def2-TZVP level.

1.5 Characterization of QDs

A Hitachi HT7800 transmission electron microscope (TEM) was used for the acquisition of TEM images. The absorption and photoluminescence were carried out on the above mentioned instruments. Specifically, the steady-state PL spectra was excited with 808 laser (MDL-III-808-2W) and 1208 laser (MDL-H-1208-1.5W), and collected by lnGaAs detector, while transient PL spectra was excited with pulsed 808 nm laser modulated by PM-1 Laser Modulator Box and detected by a near-infrared PMT detector. Temperature-dependent photoluminescence spectra were realized by a

temperature control system (DN-1-B) made from Oxford Instruments NanoScience. Measurements were performed in toluene at 1 μM.

1.6 Characterization of annihilators

The absorption and photoluminescence were performed on the previously mentioned instruments. Specifically, the excitation spectra were detected at 554 nm for Rubrene and 543 nm for TIPS-Tc with xenon lamp. Temperature-dependent fluorescence spectra were measured using a temperature control system under 600 nm excitation. Measurements were performed in toluene. The IR spectra were measured using an fourier transform infrared spectrometer (Nicolet iS50). Calculations were performed with Gaussian 09 based on the B3LYP/def2-TZVP level in toluene.

2. Supplementary Figures

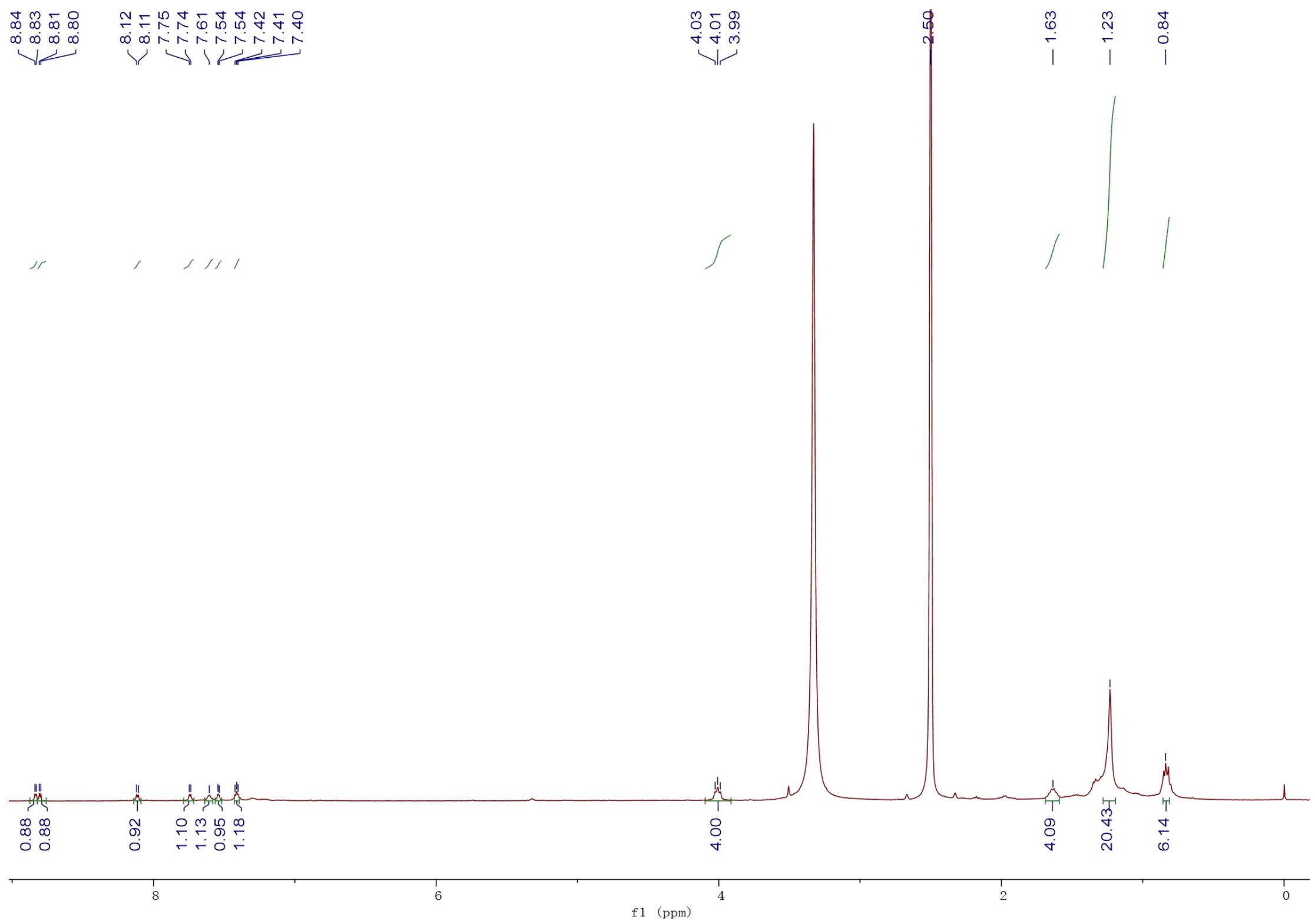

Fig. S1. ^{1}H NMR (400 MHz, DMSO-d_6) of NKU-0.

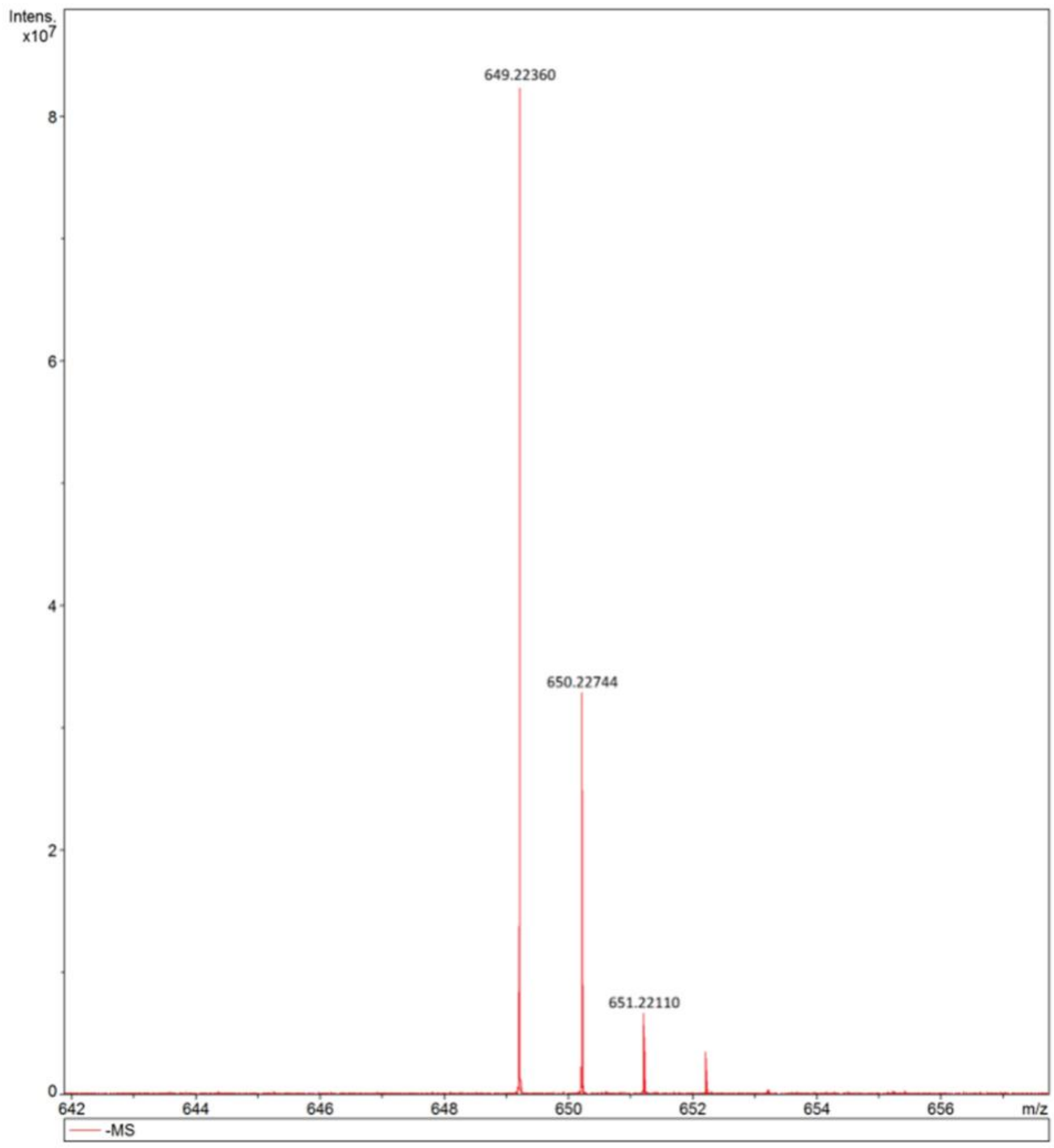

Fig. S2. High-resolution MS spectrum of NKU-0.

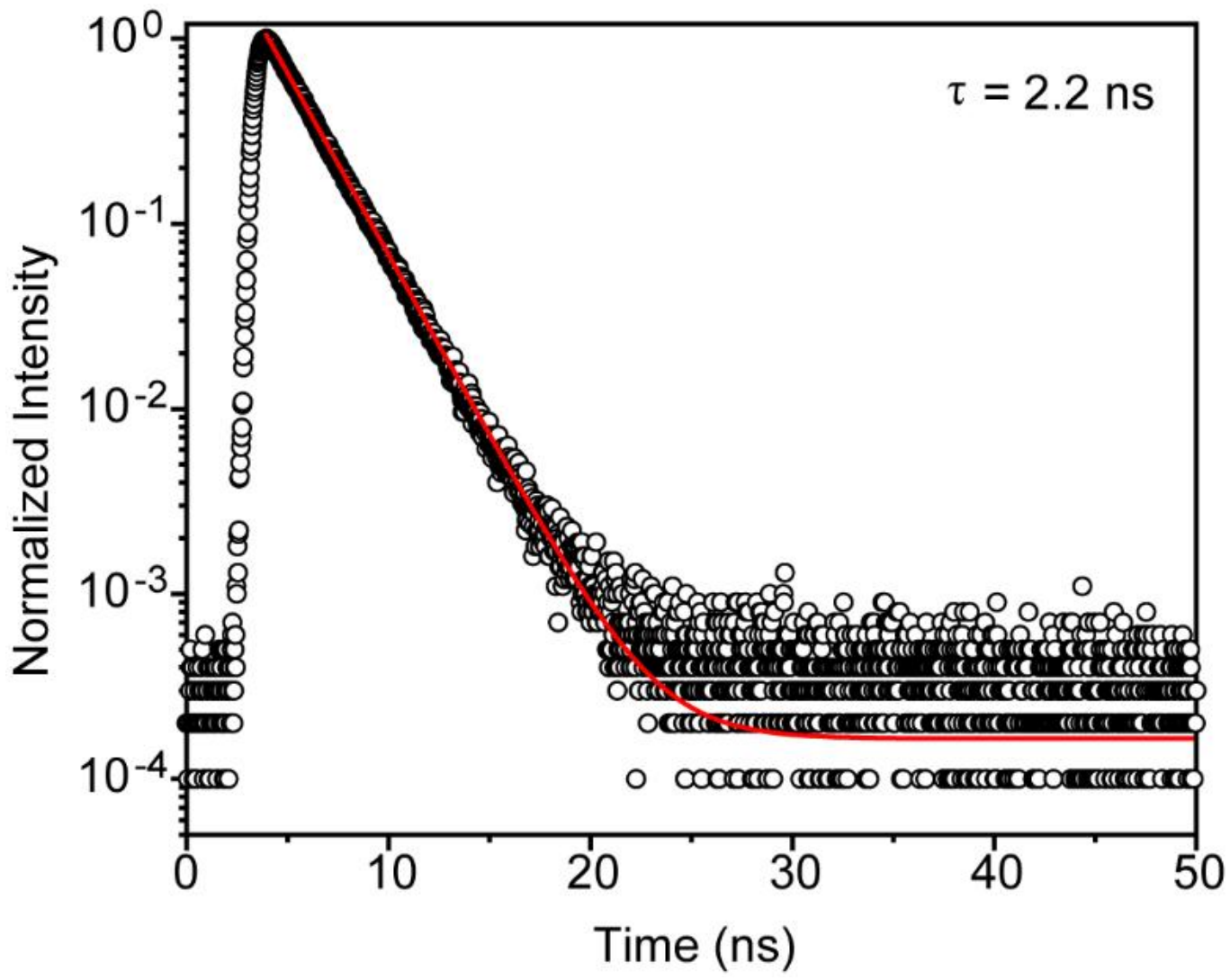

Fig. S3. Fluorescence decay traces of NKU-0. Measured in chloroform, 10 μM, λ_{ex} = 405 nm.

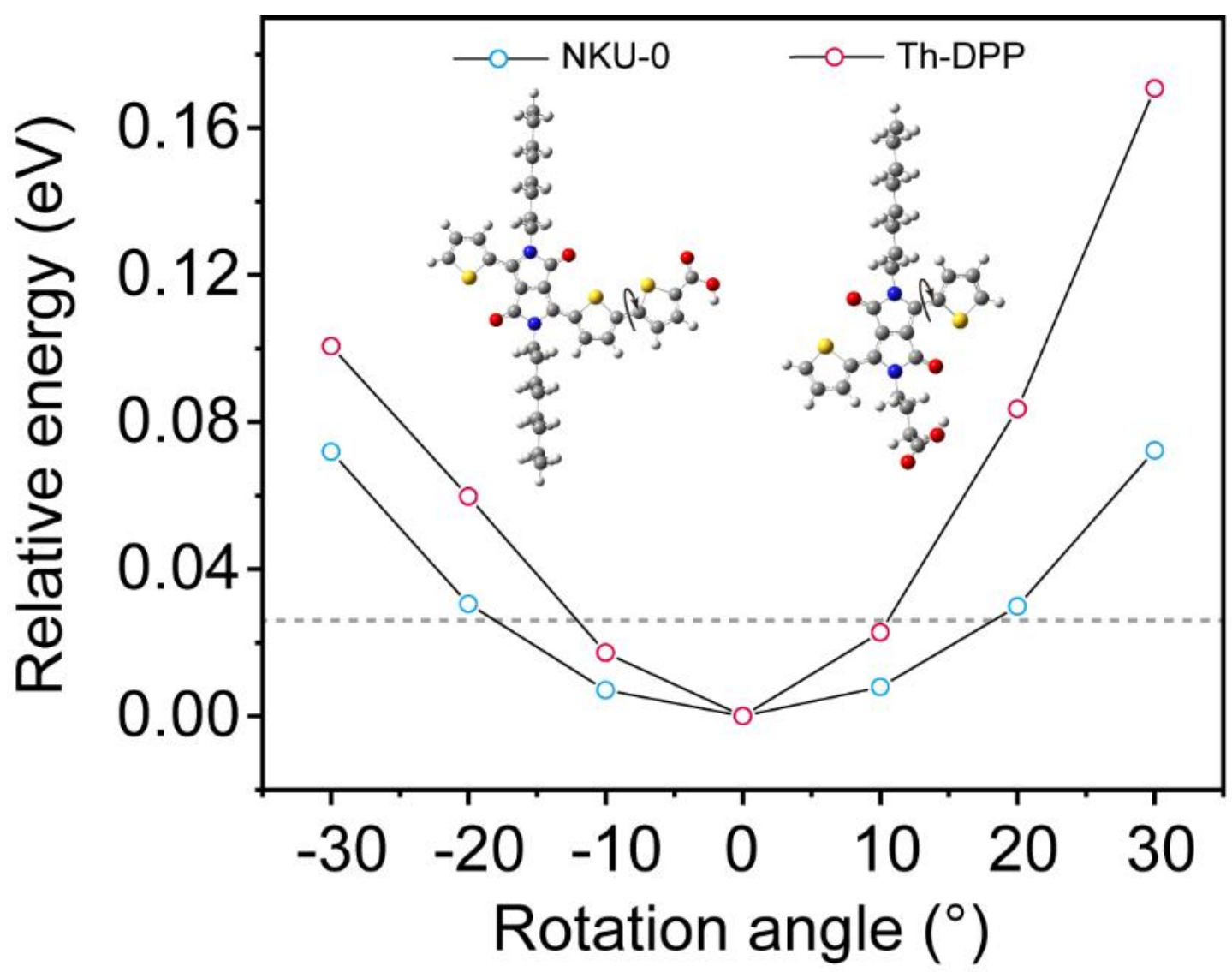

Fig. S4. The potential energy surface scans of the thiophene ring in Th-DPP and the extended thiophene ring in NKU-0 at the optimized S_1 configuration. The dotted line represents room temperature energy (26 meV).

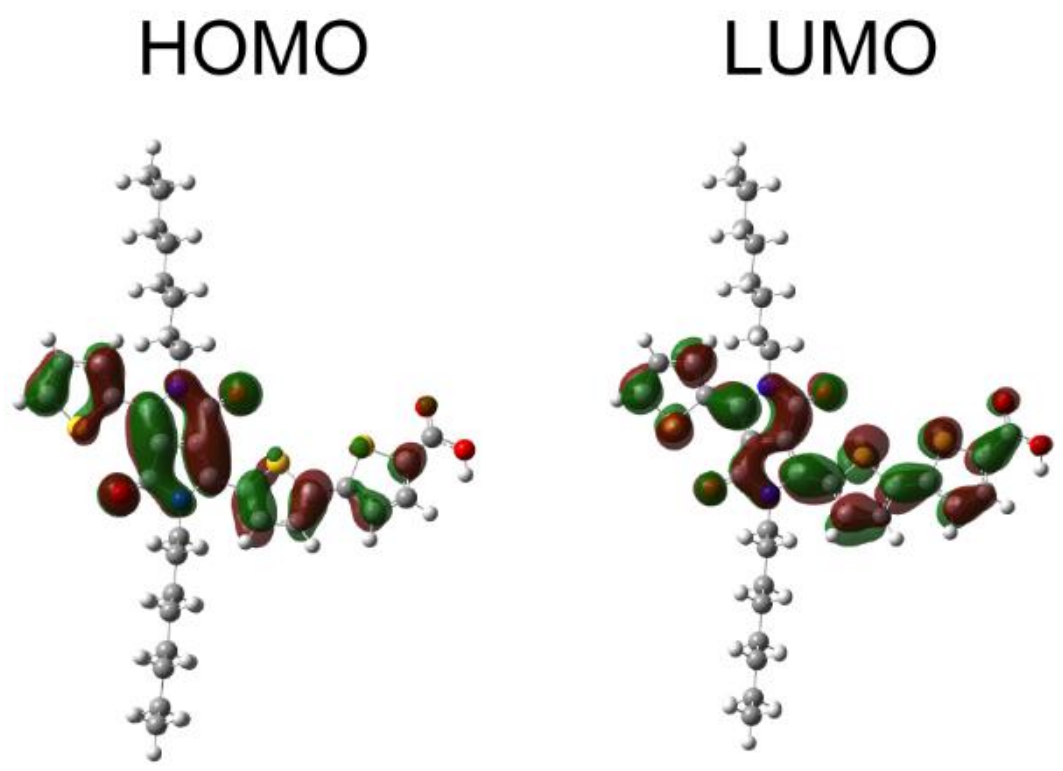

Fig. S5. Selected frontier molecular orbitals of NKU-0.

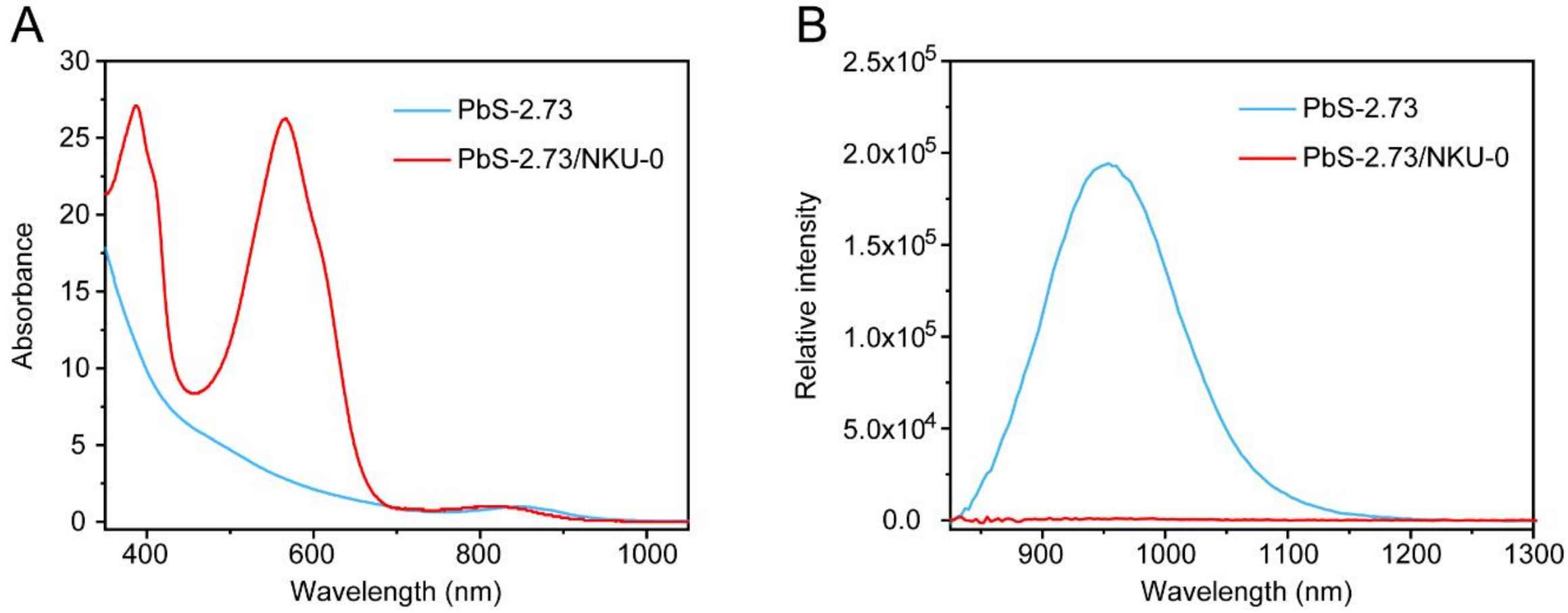

Fig. S6. Effect of NKU-0 on PbS-2.73. (A) The absorption spectra and (B) relative photoluminescence intensity of PbS-2.73 before and after ligand exchange with 500 μM NKU-0.

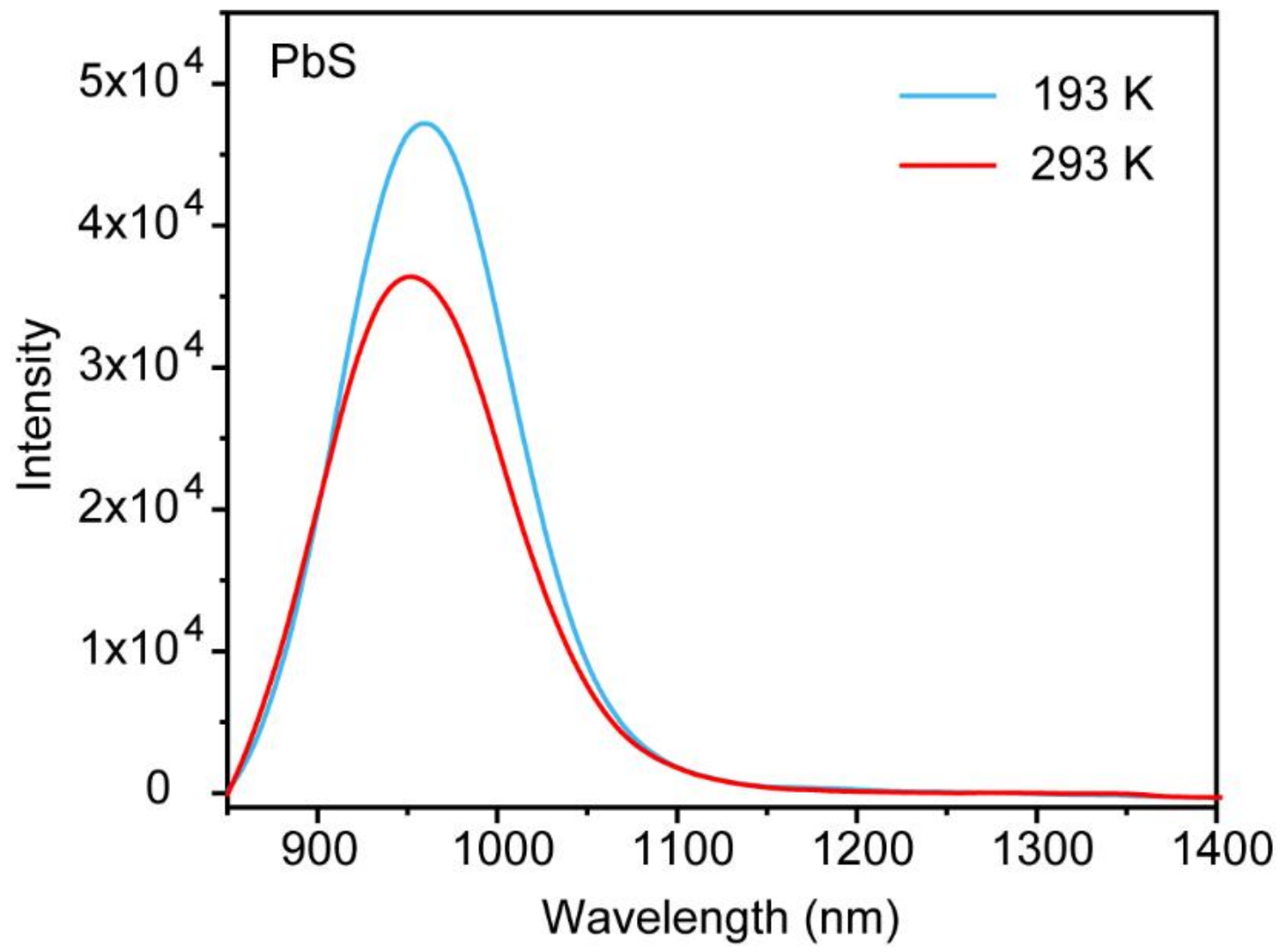

Fig. S7. Photoluminescence spectra of PbS-2.73 at 193 K and 293 K.

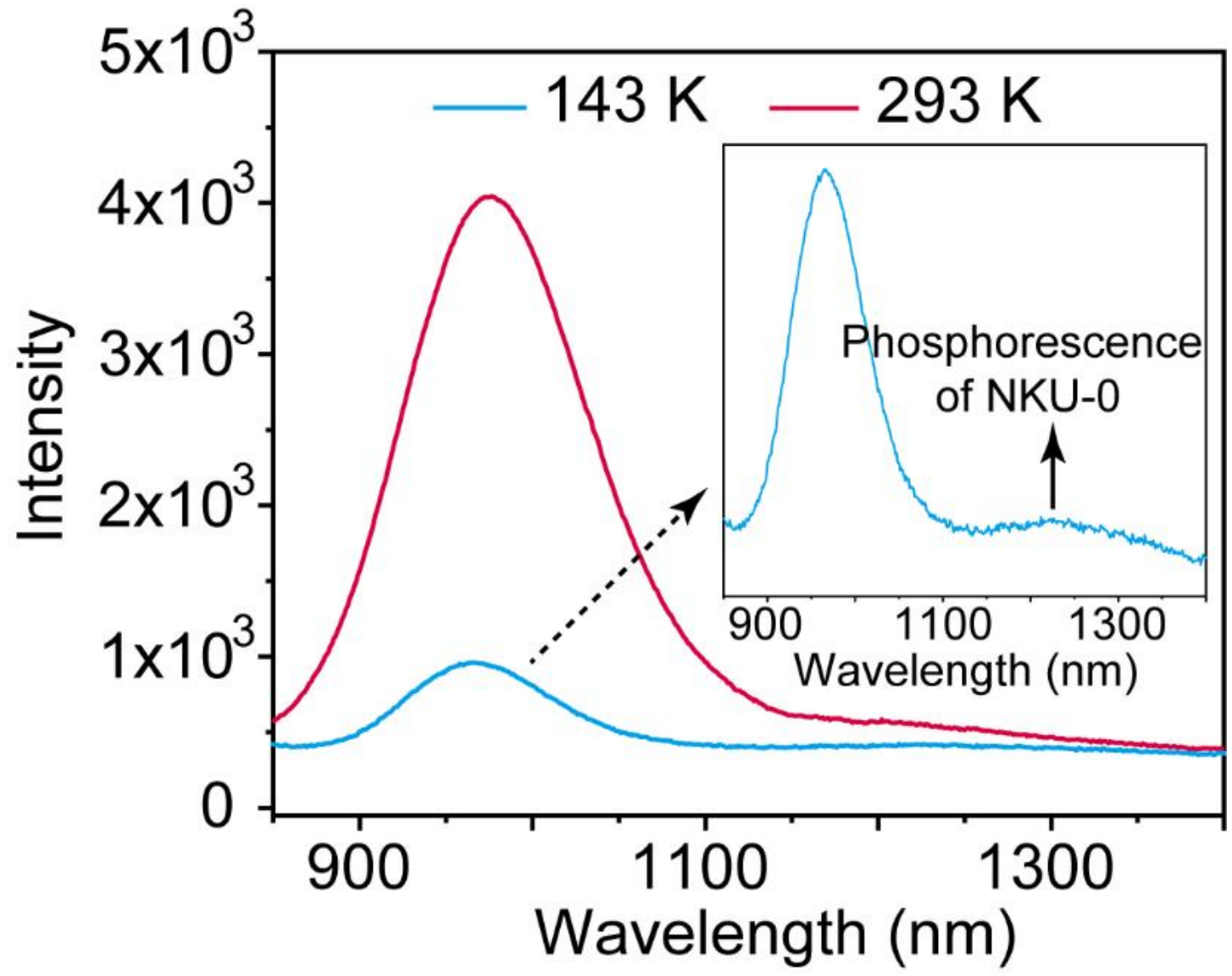

Fig. S8. Photoluminescence spectra of PbS-2.73/NKU-0 at 143 K and 293 K. The spectrum recorded at 143 K is magnified to clearly observe the phosphorescence of NKU-0.

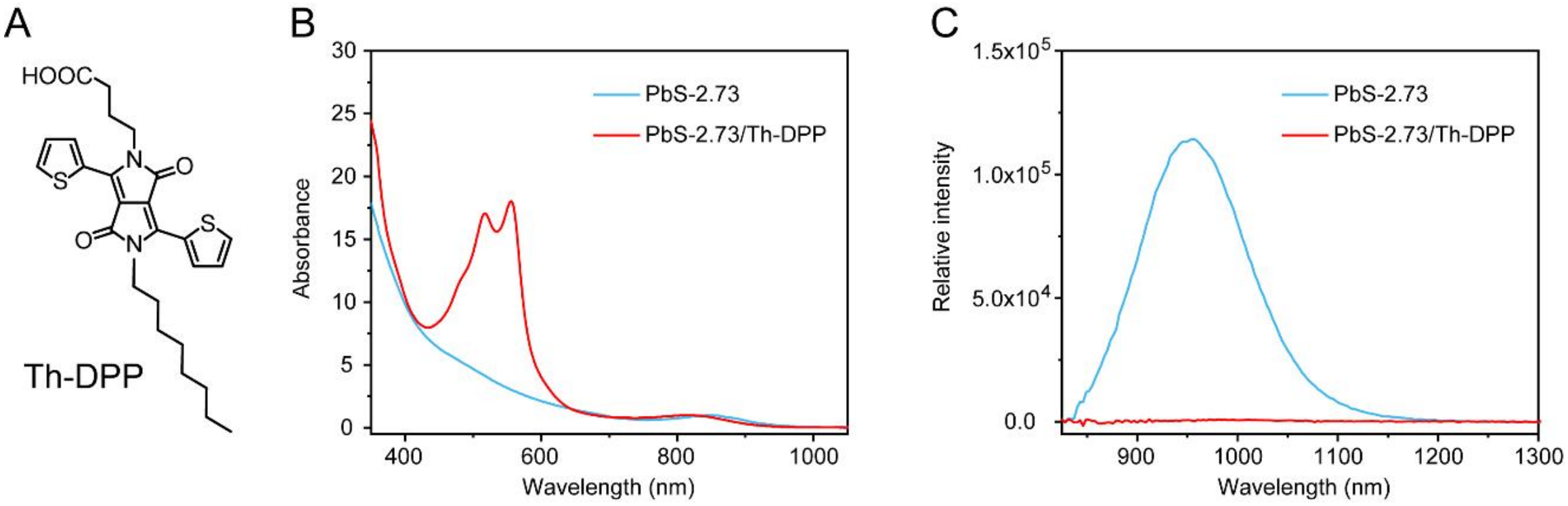

Fig. S9. Effect of Th-DPP on PbS-2.73. (A) The structure of Th-DPP. (B) The absorption spectra and (C) relative photoluminescence intensity of PbS-2.73 before and after ligand exchange with 500 μM Th-DPP.

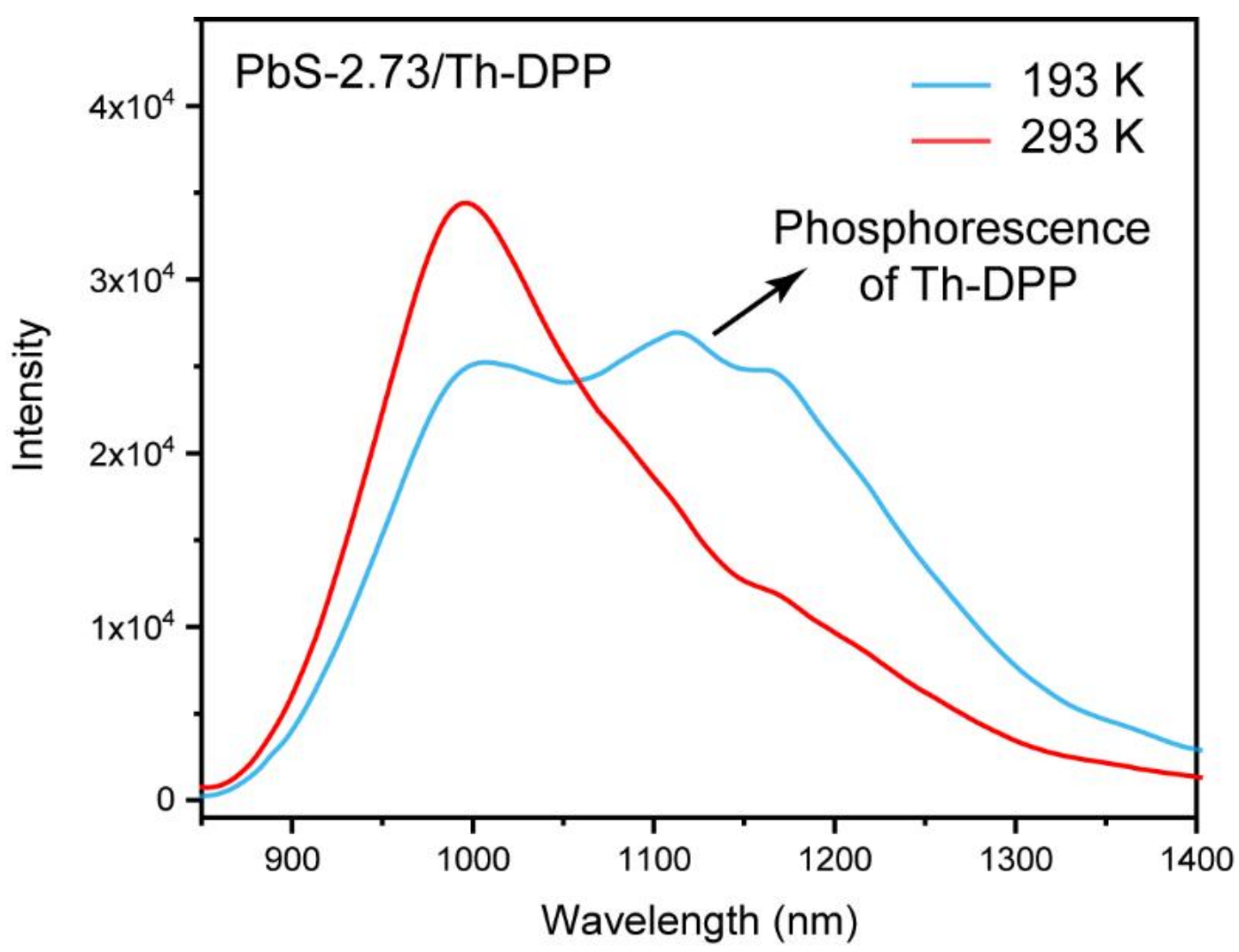

Fig. S10. Photoluminescence spectra of PbS-2.73/Th-DPP at 193 K and 293 K.

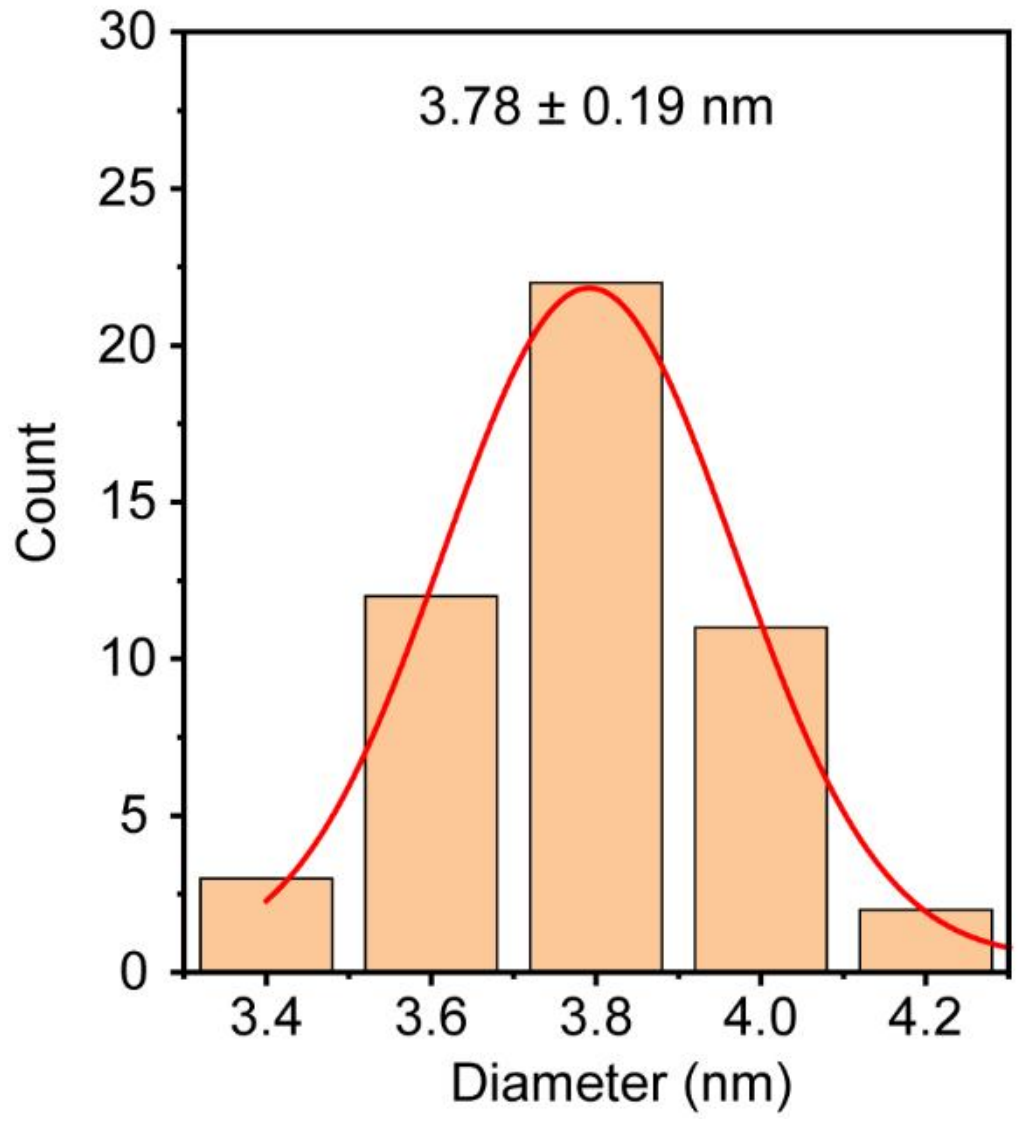

Fig. S11. The size distribution histogram of PbS-3.76 and gaussian fit.

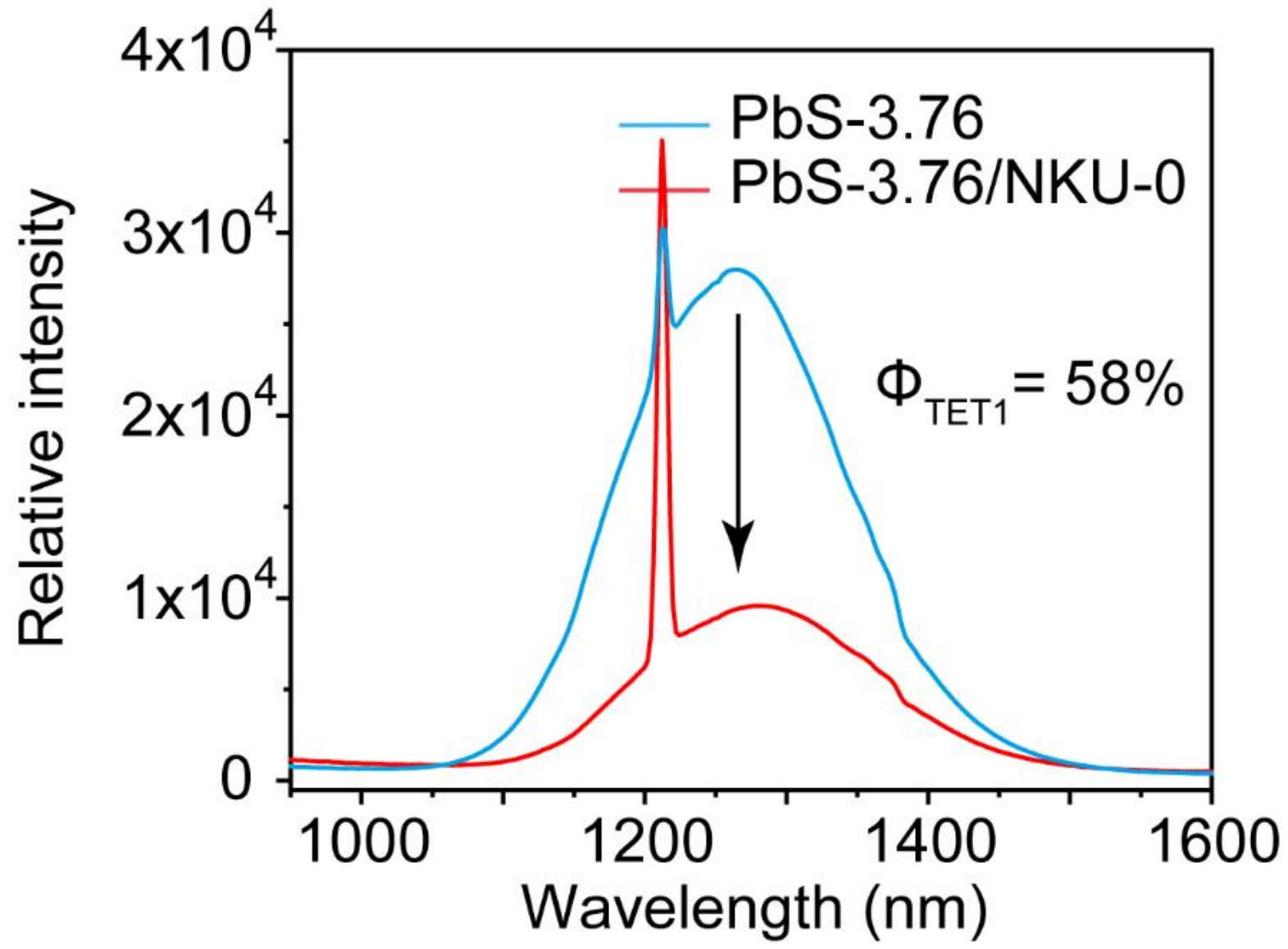

Fig. S12. Relative photoluminescence intensities of PbS-3.76 and PbS-3.76/NKU-0. Measured in argon-saturated toluene under 1208 nm excitation.

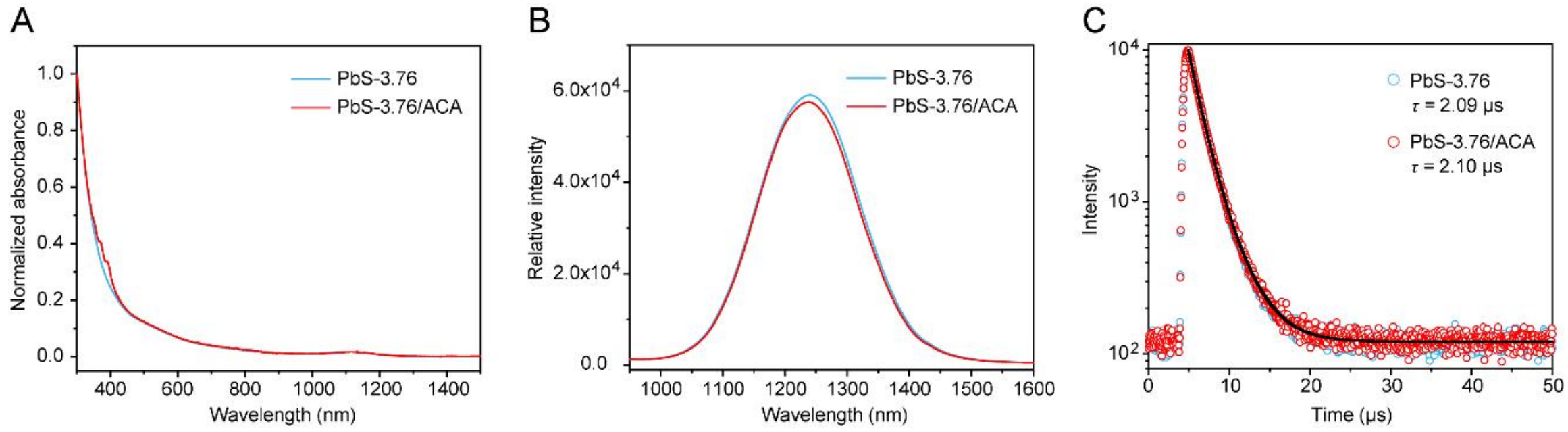

Fig. S13. Effect of ACA on PbS-3.76. (A) The absorption spectra of PbS-3.76 with and without 9-anthracenecarboxylic acid (ACA) ligands. (B) Relative photoluminescence intensities and (C) time-resolved photoluminescence spectra of PbS-3.76 and PbS-3.76/ACA in argon-saturated toluene. Measurements were performed at 1 μM, λ_{ex} = 808 nm.

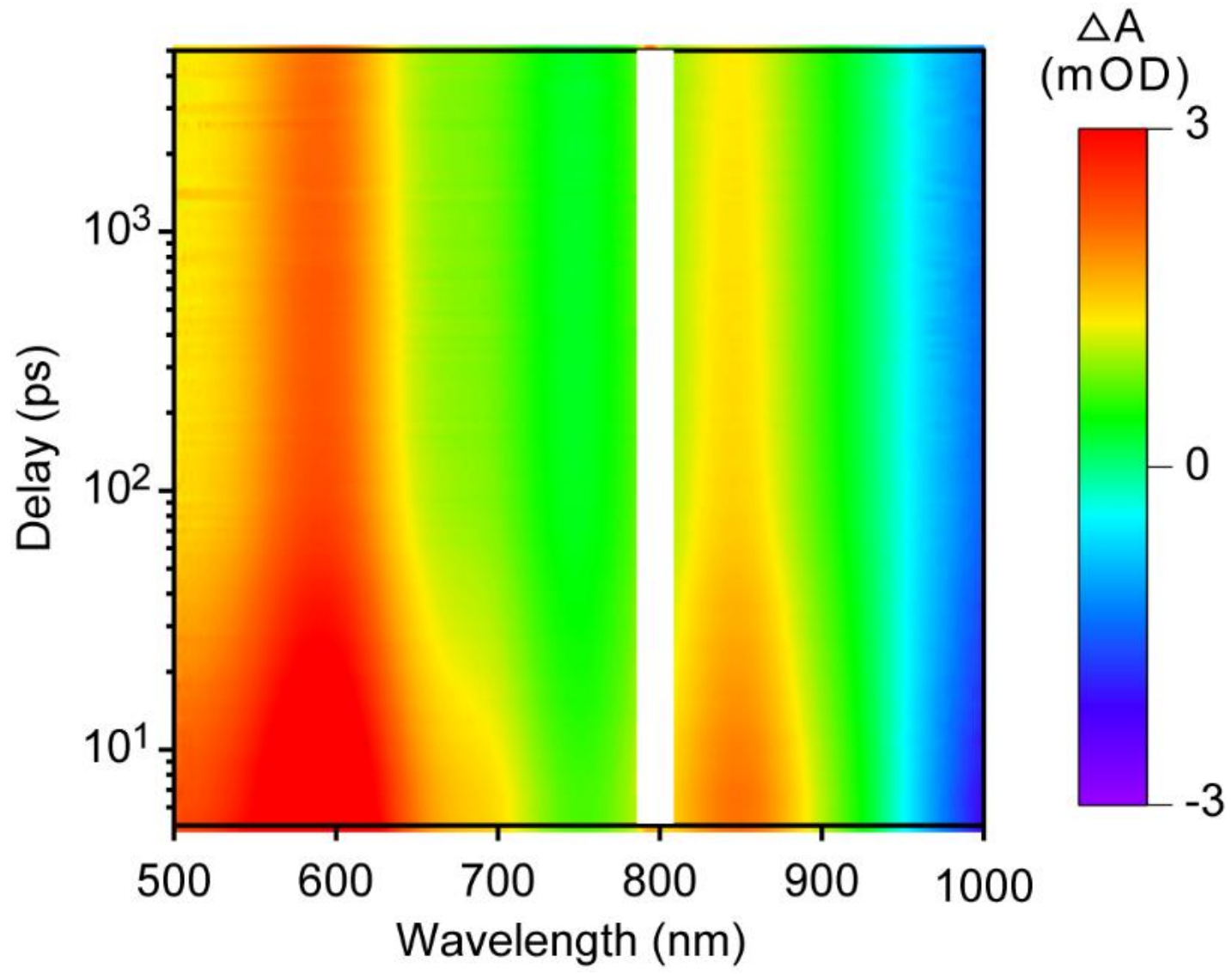

Fig. S14. Femtosecond transient absorption analysis of PbS-3.76. Two-dimensional pseudo-colour femtosecond transient absorption spectra of PbS-3.76 following 800 nm excitation.

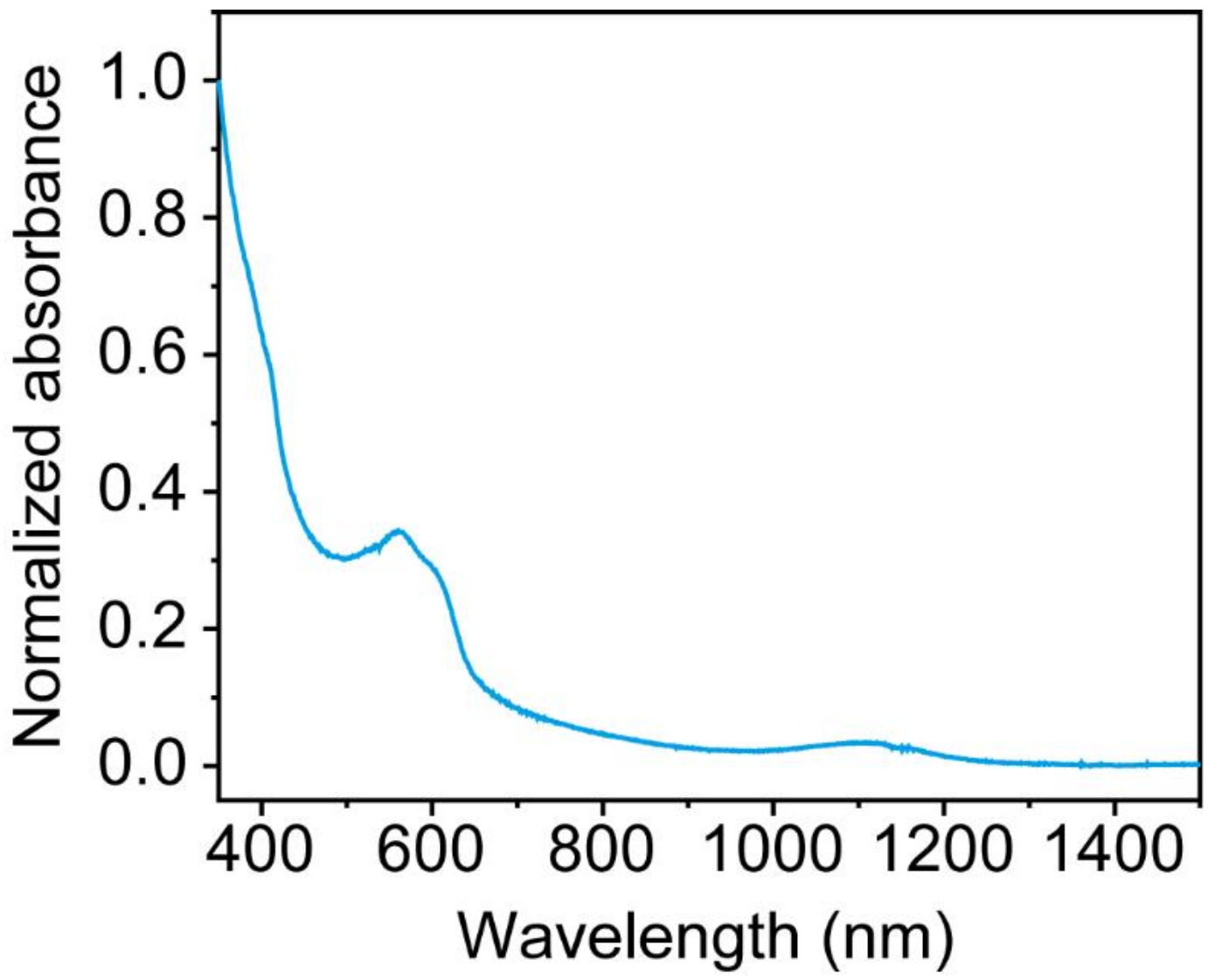

Fig. S15. The absorption spectrum of PbS-3.76/NKU-0'. PbS-3.76/NKU-0' represents the material with low NKU-0 loading on the surface of PbS-3.76.

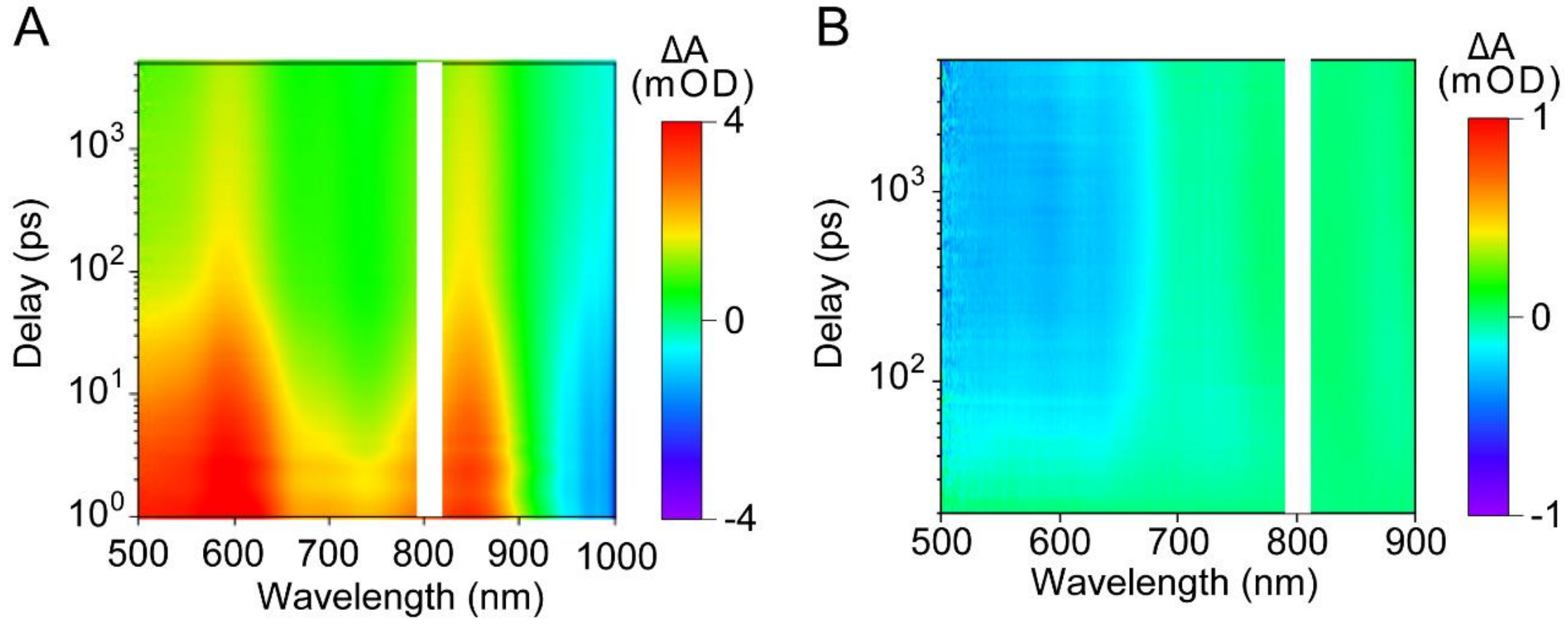

Fig. S16. Femtosecond transient absorption analysis of PbS-3.76/NKU-0'. (A) Two-dimensional pseudo-colour femtosecond transient absorption (fs-TA) spectra of PbS-3.76/NKU-0' following 800 nm excitation. (B) The double-difference fs-TA spectra after subtracting QDs contribution from PbS-3.76/NKU-0'.

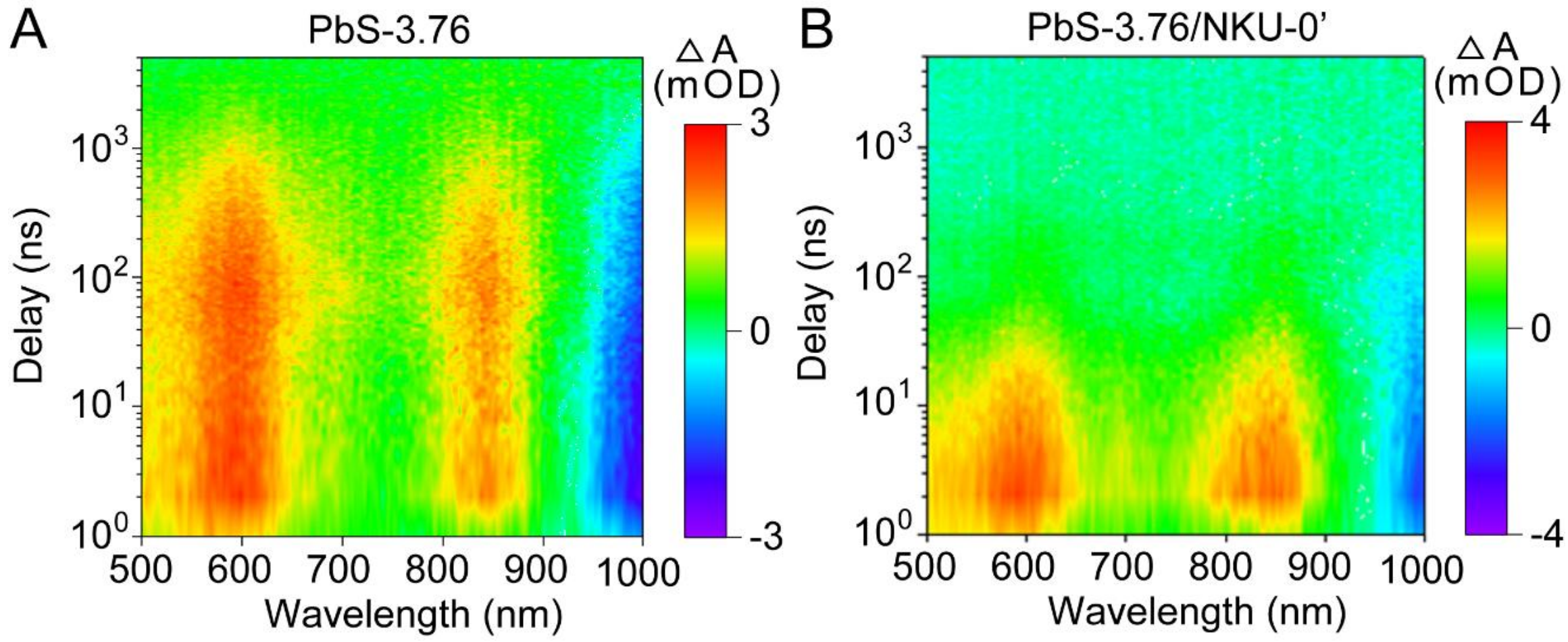

Fig. S17. Nanosecond transient absorption analysis. Two-dimensional pseudo-colour nanosecond transient absorption (ns-TA) spectra of (A) PbS-3.76 and (B) PbS-3.76/NKU-0’ within 5 μs following 800 nm excitation.

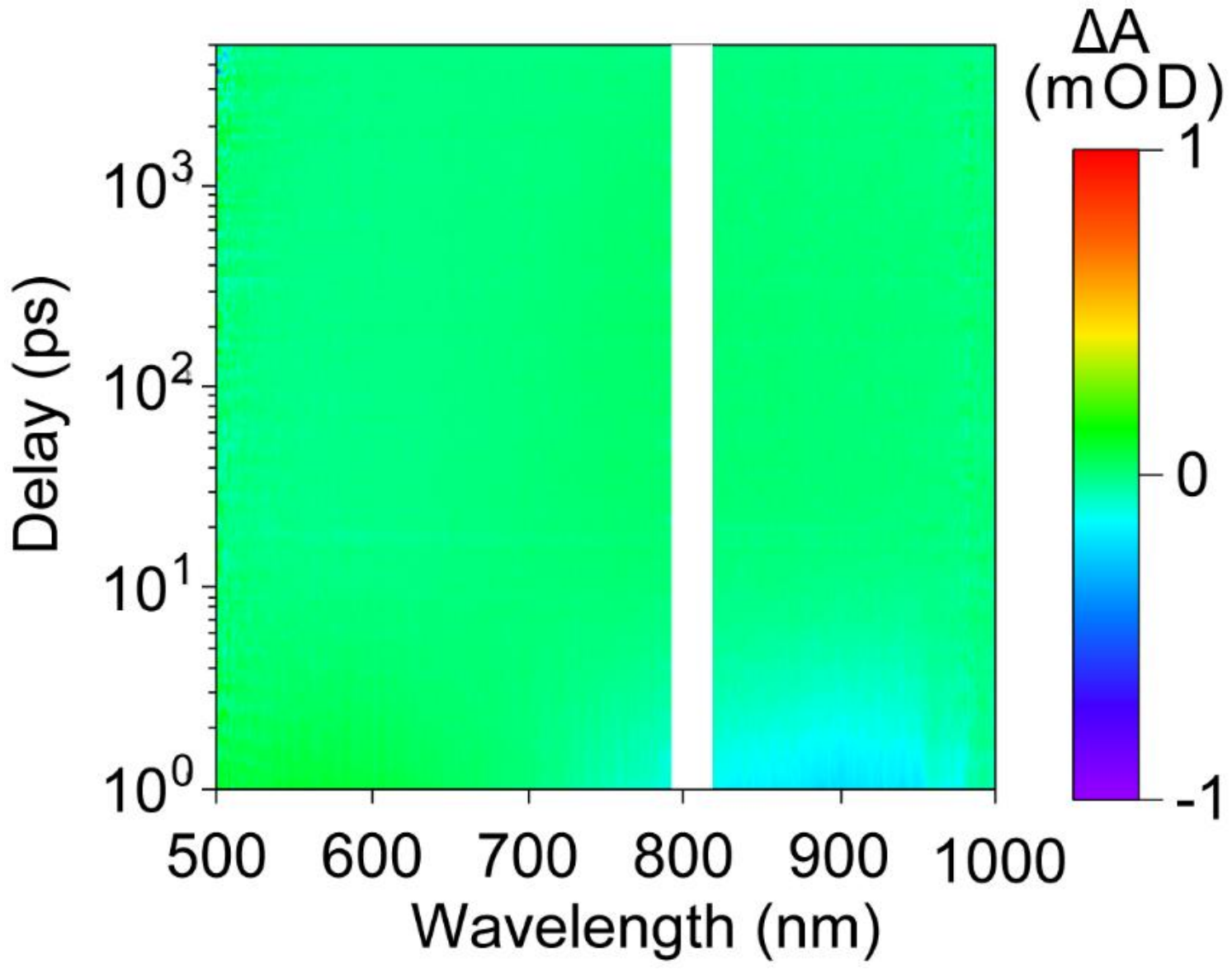

Fig. S18. Femtosecond transient absorption analysis of NKU-0. Two-dimensional pseudo-colour femtosecond transient absorption (fs-TA) spectra of NKU-0 following 800 nm excitation.

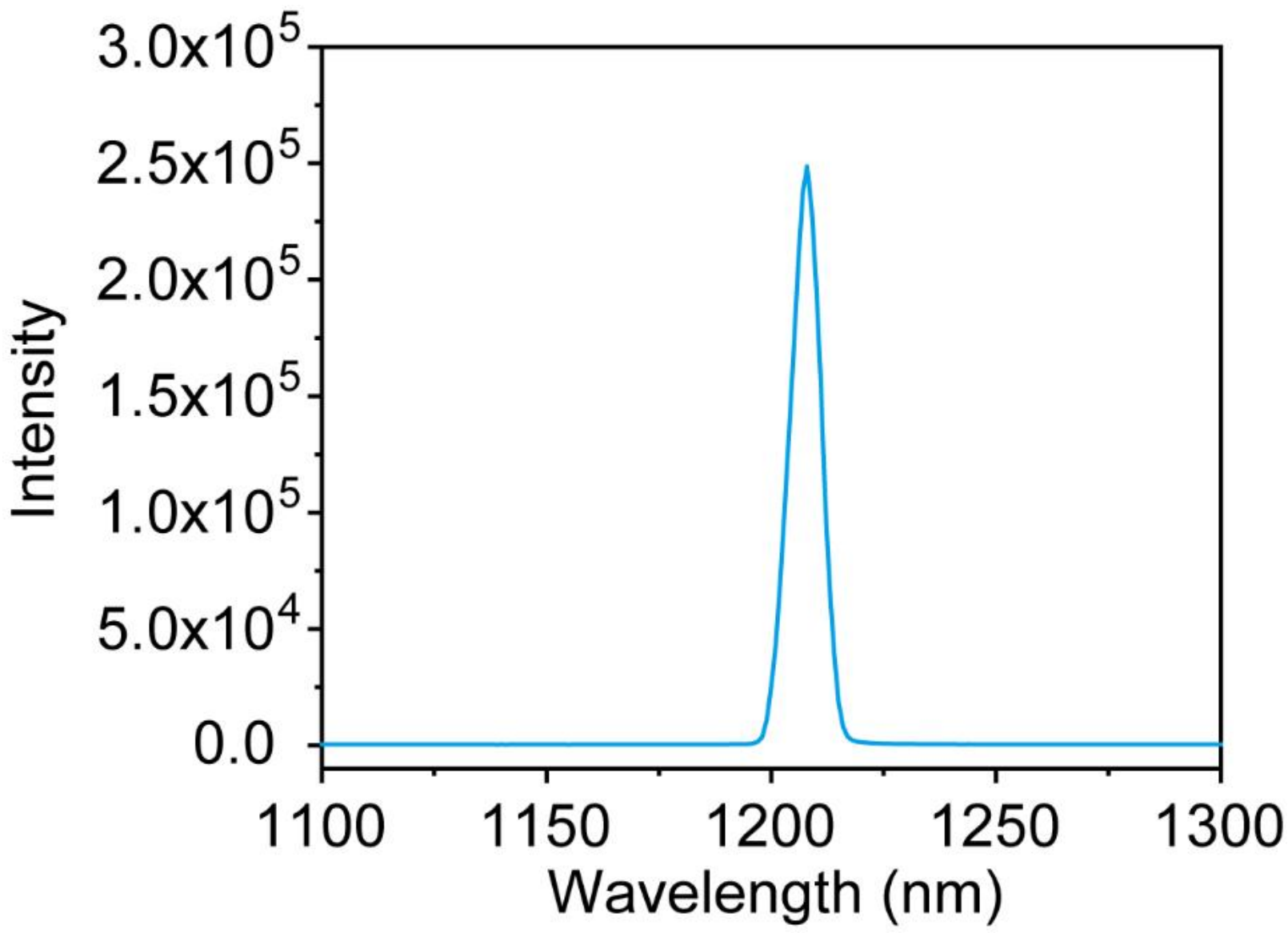

Fig. S19. The spectrum of the 1208 nm excitation source.

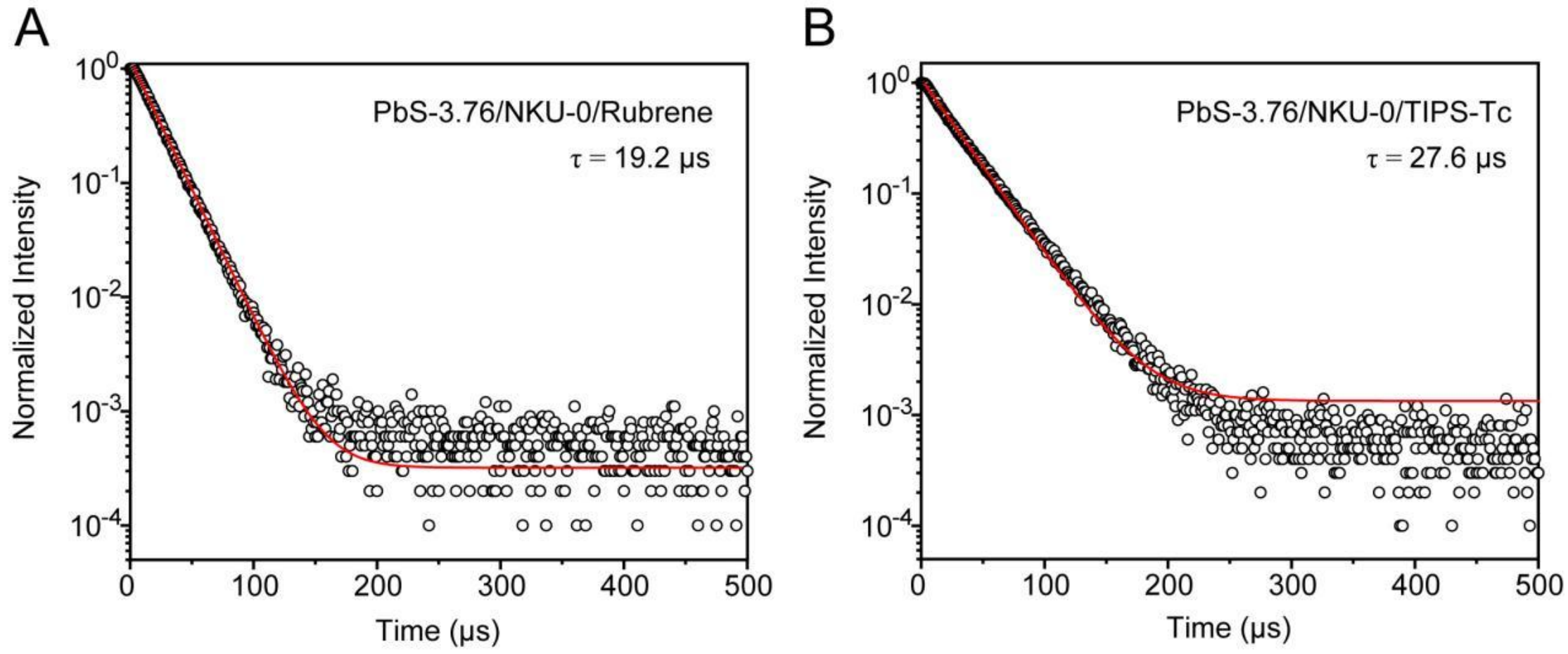

Fig. S20. Time-resolved TFU spectra. (A) PbS-3.76/NKU-0/Rubrene. (B) PbS-3.76/NKU-0/TIPS-Tc.

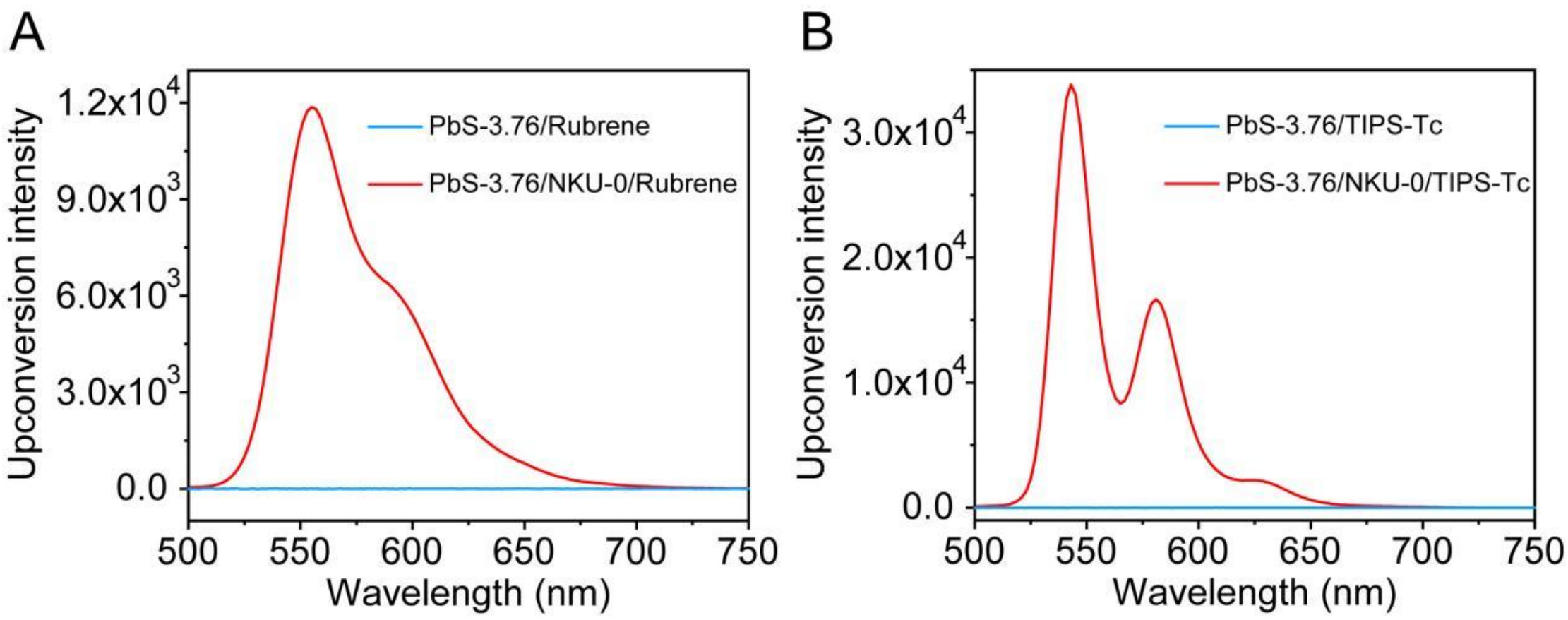

Fig. S21. Effect of the presence or absence of the NKU-0 ligand on the upconversion systems. The systems employ (A) Rubrene and (B) TIPS-Tc as annihilators, respectively.

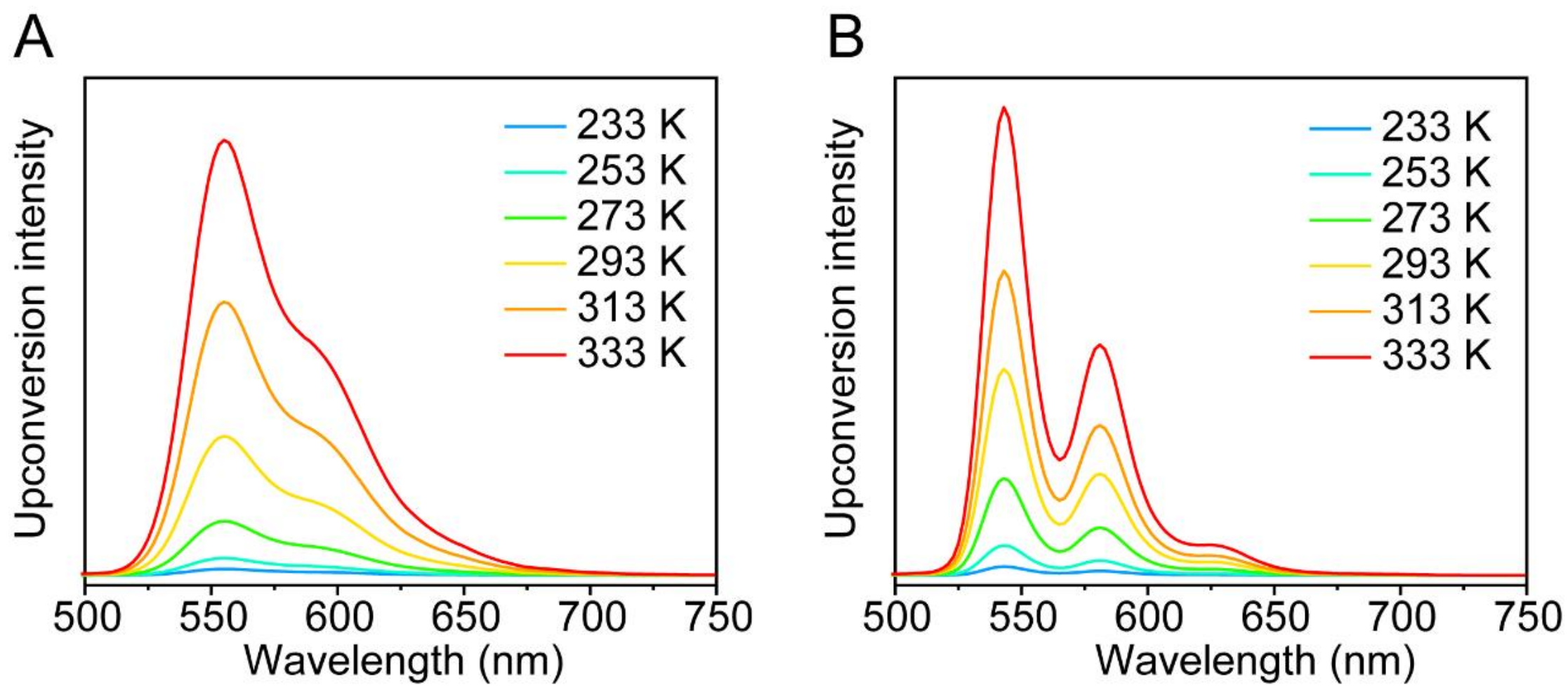

Fig. S22. Temperature-dependent upconversion measurements. The measurements were performed on (A) PbS-3.76/NKU-0/Rubrene and (B) PbS-3.76/NKU-0/TIPS-Tc under 1208 nm excitation.

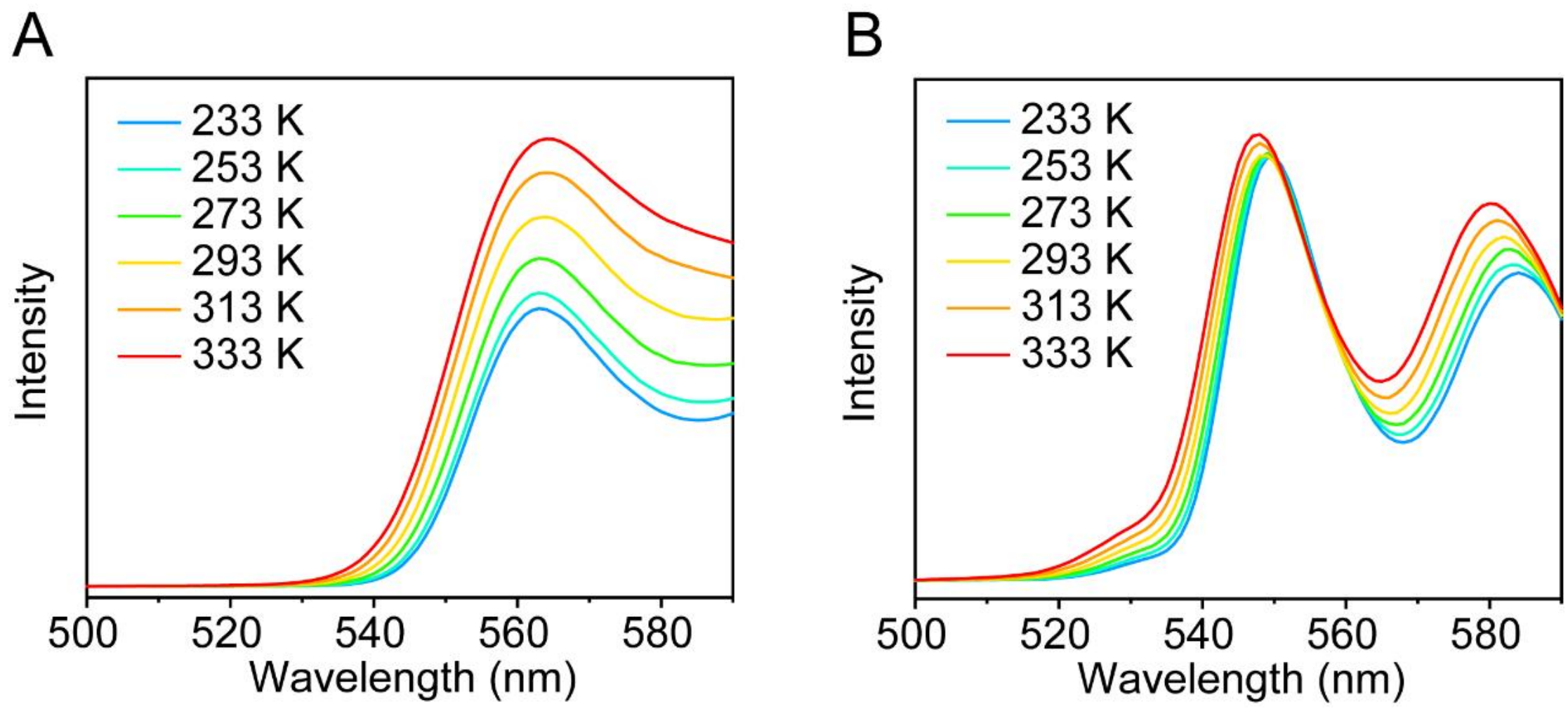

Fig. S23. Temperature-dependent fluorescence measurements. The measurements were performed on (A) Rubrene and (B) TIPS-Tc under 600 nm excitation. Due to the influence of the excitation light, the spectra are recorded only up to 590 nm.

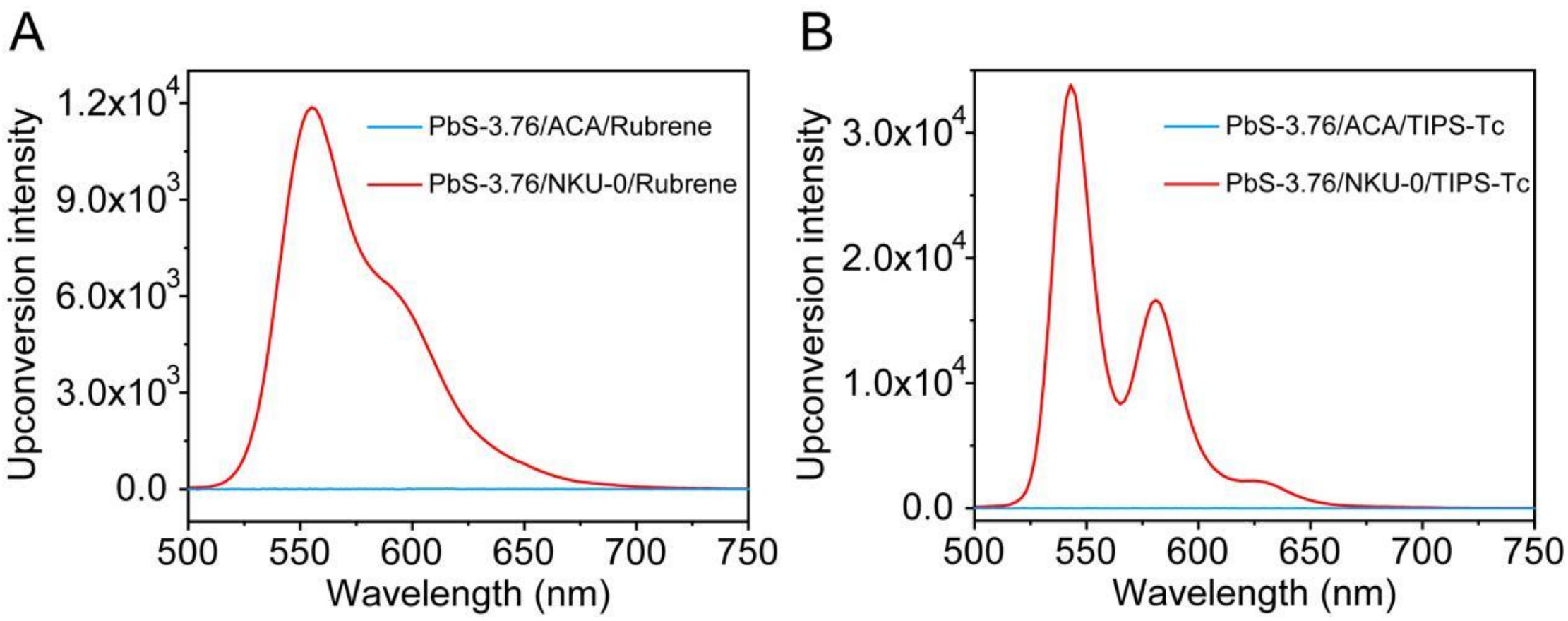

Fig. S24. Upconversion measurements of PbS-3.76/ACA or PbS-3.76/NKU-0 as photosensitizers. The systems employ (A) Rubrene and (B) TIPS-Tc as annihilators, respectively.

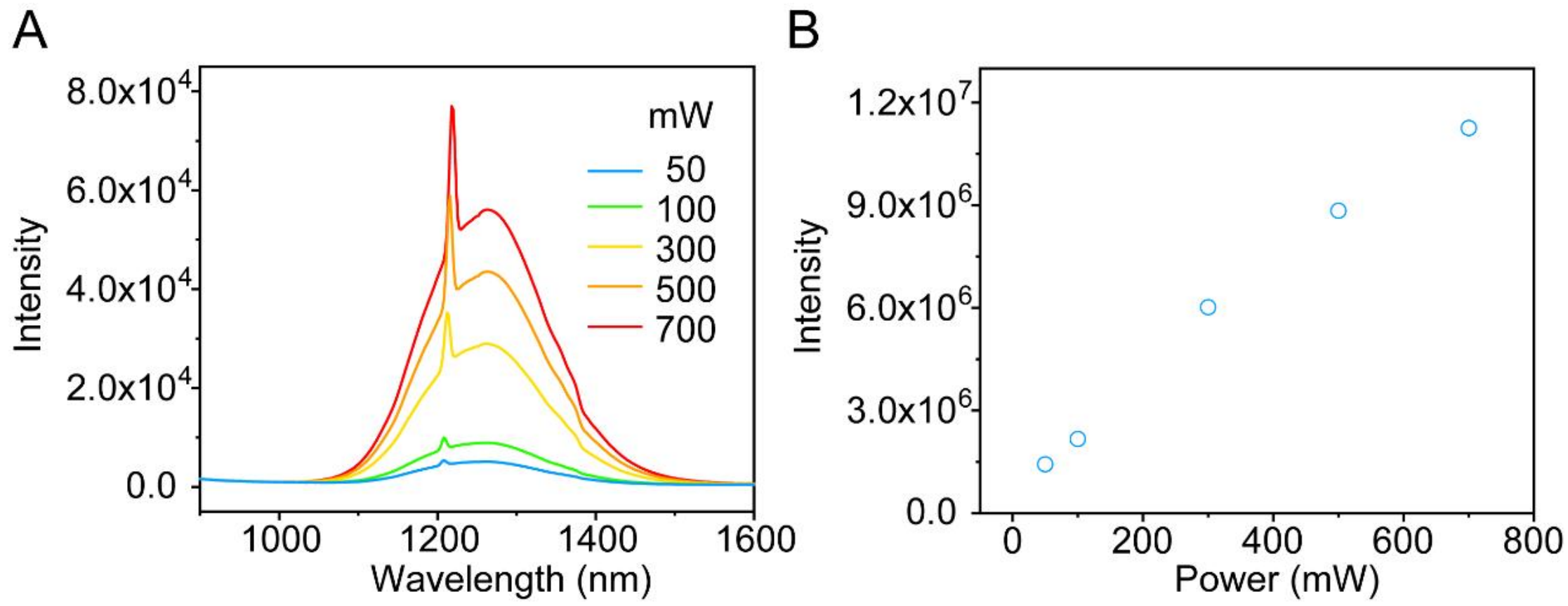

Fig. S25. Power-dependent photoluminescence of PbS-3.76. (A) Photoluminescence spectra at different excitation powers under 1208 nm excitation. (B) The dependence of the PbS-3.76 photoluminescence intensity on the excitation power.

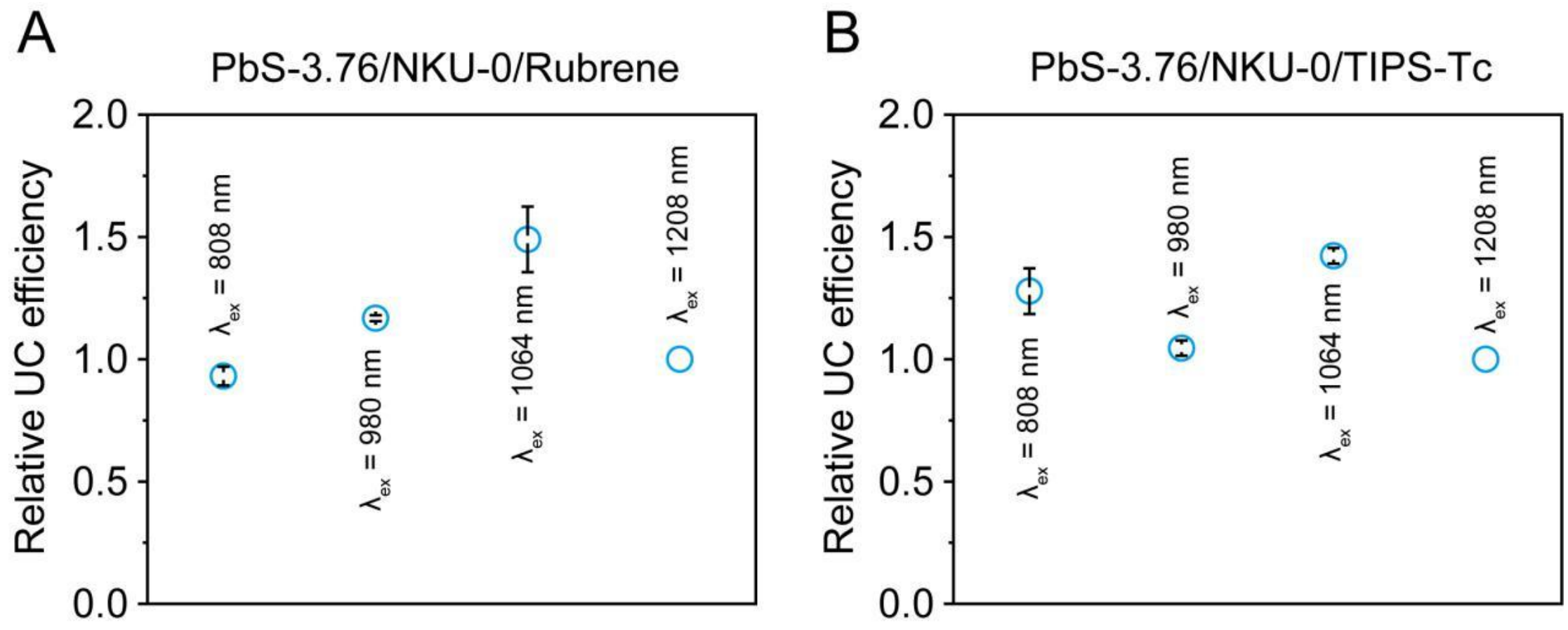

Fig. S26. Relative upconversion efficiency at different excitation wavelengths. The measurements were performed on (A) PbS-3.76/NKU-0/Rubrene and (B) PbS-3.76/NKU-0/TIPS-Tc, with 1208 nm excitation as a reference.

3. Supplementary Tables

Table S1. Photophysical properties of NKU-0. [a]

	λ_{abs} [b]	ε [c]	λ_{em} [d]	S_1 [e]	T_1 [f]	Φ [g]	τ_f [h]
NKU-0	560	2.32	625	2.0	1.0	22	2.2

[a] Measured in chloroform, 10 μM. [b] Maximum absorption wavelength (nm). [c] Molar absorption coefficient (×10^4 $M^{-1}cm^{-1}$). [d] Maximum fluorescence emission wavelength (nm). [e] The S_1 energy levels (eV) was determined by the intersection of absorption and emission. [f] The T_1 energy levels (eV) was determined by the phosphorescence of NKU-0 sensitized by QDs. [g] Absolute fluorescence quantum yield was determined with an integrating sphere (%). [h] Fluorescence lifetime (ns).

Table S2. Photophysical properties of PbS-3.76 QDs. [a]

	λ_{abs} [b]	bandgap [c]	ε [d]	λ_{em} [e]	PLQY [f]
PbS-3.76	1113	1.0	7.43	1237	62

[a] Measured in toluene, 1 μM. [b] The first exciton absorption peak (nm). [c] The bandgap (eV) was determined using the Tauc Plot method. [d] Molar absorption coefficient at the first exciton absorption peak ($\times 10^4$ $M^{-1}cm^{-1}$) was calculated on the basis of particle size. [e] Maximum emission wavelength (nm). [f] The absolute photoluminescence quantum yield (%) was determined with an integrating sphere.

Table S3. The fitting results of bleach recovery kinetics.

	τ_1 [a]	τ_2 [a]	τ_3 [a]	τ_4 [b]
PbS-3.76	17.3	76.7	lnf	--
PbS-3.76/NKU-0	17.3	76.7	lnf	780.5

[a] The time component of PbS-3.76 intrinsic decay as global parameters (ps). [b] The time component of triplet exciton transfer process (ps, $k_{TET1} = 1/\tau_4$).

Table S4. Summary of reported triplet fusion upconversion with excitation wavelength exceeding 700 nm.

Photosensitizer	Annihilator	λ_{ex} / nm [a]	Anti-Stokes shift / eV	Reference
PdTNP	Py5	720 (1.72)	0.45 (-1.27) [b]	(*56*)
$Os(peptpy)_2^{2+}$	TTBP	724 (1.71)	0.97 (-0.74) [b]	(*57*)
TMD	Rubrene/DBP	772 (1.61)	0.43 (-1.18) [b]	(*58*)
Pyr_1RuPZn_2	PDI<br>tetracene	780 (1.59)	0.70 (-0.89) [b]<br>0.86 (-0.73) [b]	(*59*)
PbS/5-CT	Rubrene	781 (1.59)	0.63 (-0.96) [a]	(*35*)
$PtAg_{24}(SR)_{18}$	TIPS-Ac	785 (1.58)	1.04 (-0.54) [b]	(*60*)
PbS/CdS/5-CT	Rubrene	785 (1.58)	0.63 (-0.95) [b]	(*52*)
PbS/CdS/5-CT	Rubrene	808 (1.53)	0.68 (-0.85) [b]	(*61*)
ZCISe/TCA	Rubrene	808 (1.53)	0.7 (-0.83) [b]	(*62*)
InAs/ZnSe/5-CT	Rubrene	808 (1.53)	0.64 (-0.89) [b]	(*63*)
Y6	Rubrene	850 (1.46)	0.74 (-0.72) [b]	(*64*)
Os-lipophilic	Rubrene	938 (1.32)	0.86 (-0.46) [b]	(*16*)
PbS	TES-ADT	975 (1.27)	0.76 (-0.51) [b]	(*65*)
Yb-L	Rubrene	980 (1.27)	0.95 (-0.32) [b]	(*66*)
PbSe	Rubrene	980 (1.27)	0.92 (-0.35) [b]	(*67*)
PbS	TES-ADT	1064 (1.17)	0.86 (-0.31) [b]	(*24*)
PbS-3.11/Th-DPP	Rubrene	1064 (1.17)	1.07 (-0.10) [b]	(*25*)
PbS/TTCA	V79	1140 (1.09)	0.68 (-0.41) [b]	(*4*)
**PbS/NKU-0**	**Rubrene**<br>**TIPS-Tc**	**1208 (1.03)**	**1.21 (+0.18)** [b]<br>**1.26 (+0.23)** [b]	**This work**

[a] Excitation wavelength (nm), where the value in parentheses denotes the photon energy of a single excitation photon (eV). [b] The difference between the anti-Stokes shift and the energy of a single excitation photon for the corresponding photon-upconversion material (eV). The negative value corresponds to the anti-Stokes shift below this energy, while the positive value corresponds to the anti-Stokes shift above it.

REFERENCES

1. J. Zhou, Q. Liu, W. Feng, Y. Sun, F. Li, Upconversion luminescent materials: Advances and applications. *Chem. Rev.* **115**, 395–465 (2015).

2. L. Zeng, L. Huang, J. Han, G. Han, Enhancing triplet–triplet annihilation upconversion: From molecular design to present applications. *Acc. Chem. Res.* **55**, 2604–2615 (2022).

3. X. Cheng, J. Zhou, J. Yue, Y. Wei, C. Gao, X. Xie, L. Huang, Recent development in sensitizers for lanthanide-doped upconversion luminescence. *Chem. Rev.* **122**, 15998–16050 (2022).

4. E. M. Gholizadeh, S. K. K. Prasad, Z. L. Teh, T. Ishwara, S. Norman, A. J. Petty, J. H. Cole, S. Cheong, R. D. Tilley, J. E. Anthony, S. Huang, T. W. Schmidt, Photochemical upconversion of near-infrared light from below the silicon bandgap. *Nat. Photonics* **14**, 585–590 (2020).

5. T. F. Schulze, T. W. Schmidt, Photochemical upconversion: Present status and prospects for its application to solar energy conversion. *Energ. Environ. Sci.* **8**, 103–125 (2015).

6. B. D. Ravetz, A. B. Pun, E. M. Churchill, D. N. Congreve, T. Rovis, L. M. Campos, Photoredox catalysis using infrared light via triplet fusion upconversion. *Nature* **565**, 343–346 (2019).

7. L. Huang, G. Han, Triplet–triplet annihilation photon upconversion-mediated photochemical reactions. *Nat. Rev. Chem.* **8**, 238–255 (2024).

8. S. N. Sanders, T. H. Schloemer, M. K. Gangishetty, D. Anderson, M. Seitz, A. O. Gallegos, R. C. Stokes, D. N. Congreve, Triplet fusion upconversion nanocapsules for volumetric 3D printing. *Nature* **604**, 474–478 (2022).

9. D. K. Limberg, J.-H. Kang, R. C. Hayward, Triplet–triplet annihilation photopolymerization for high-resolution 3D printing. *J. Am. Chem. Soc.* **144**, 5226–5232 (2022).

10. B. Zhou, B. Shi, D. Jin, X. Liu, Controlling upconversion nanocrystals for emerging applications. *Nat. Nanotechnol.* **10**, 924–936 (2015).

11. Q. Liu, Y. Zhang, C. S. Peng, T. Yang, L.-M. Joubert, S. Chu, Single upconversion nanoparticle imaging at sub-10 W cm^{-2} irradiance. *Nat. Photonics* **12**, 548–553 (2018).

12. F. Auzel, Upconversion and anti-stokes processes with f and d ions in solids. *Chem. Rev.* **104**, 139–174 (2004).

13. H. Li, M. Tan, X. Wang, F. Li, Y. Zhang, L. Zhao, C. Yang, G. Chen, Temporal multiplexed in vivo upconversion imaging. *J. Am. Chem. Soc.* **142**, 2023–2030 (2020).

14. T. N. Singh-Rachford, F. N. Castellano, Photon upconversion based on sensitized triplet–triplet annihilation. *Coord. Chem. Rev.* **254**, 2560–2573 (2010).

15. S. E. Seo, H.-S. Choe, H. Cho, H.-I. Kim, J.-H. Kim, O. S. Kwon, Recent advances in materials for and applications of triplet–triplet annihilation-based upconversion. *J. Mater. Chem. C* **10**, 4483–4496 (2022).

16. S. Amemori, Y. Sasaki, N. Yanai, N. Kimizuka, Near-infrared-to-visible photon upconversion sensitized by a metal complex with spin-forbidden yet strong S0–T1 absorption. *J. Am. Chem. Soc.* **138**, 8702–8705 (2016).

17. N. Yanai, N. Kimizuka, New triplet sensitization routes for photon upconversion: Thermally activated delayed fluorescence molecules, inorganic nanocrystals, and singlet-to-triplet absorption. *Acc. Chem. Res.* **50**, 2487–2495 (2017).

18. C. Mongin, S. Garakyaraghi, N. Razgoniaeva, M. Zamkov, F. N. Castellano, Direct observation of triplet energy transfer from semiconductor nanocrystals. *Science* **351**, 369–372 (2016).

19. Y. Han, S. He, K. Wu, Molecular triplet sensitization and photon upconversion using colloidal semiconductor nanocrystals. *ACS Energy Lett.* **6**, 3151–3166 (2021).

20. Y. Jiang, E. A. Weiss, Colloidal quantum dots as photocatalysts for triplet excited state reactions of organic molecules. *J. Am. Chem. Soc.* **142**, 15219–15229 (2020).

21. Z. Huang, M. L. Tang, Designing transmitter ligands that mediate energy transfer between semiconductor nanocrystals and molecules. *J. Am. Chem. Soc.* **139**, 9412–9418 (2017).

22. Z. Xu, Z. Huang, T. Jin, T. Lian, M. L. Tang, Mechanistic understanding and rational design of quantum dot/mediator interfaces for efficient photon upconversion. *Acc. Chem. Res.* **54**, 70–80 (2021).

23. S. He, Y. Han, J. Guo, K. Wu, Entropy-powered endothermic energy transfer from cspbbr3 nanocrystals for photon upconversion. *J. Phys. Chem. Lett.* **13**, 1713–1718 (2022).

24. N. Nishimura, J. R. Allardice, J. Xiao, Q. Gu, V. Gray, A. Rao, Photon upconversion utilizing energy beyond the band gap of crystalline silicon with a hybrid TES-ADT/PbS quantum dots system. *Chem. Sci.* **10**, 4750–4760 (2019).

25. L.-H. Jiang, X. Miao, M.-Y. Zhang, J.-Y. Li, L. Zeng, W. Hu, L. Huang, D.-W. Pang, Near infrared-II excited triplet fusion upconversion with anti-stokes shift approaching the theoretical limit. *J. Am. Chem. Soc.* **146**, 10785–10797 (2024).

26. P. E. Hartnett, E. A. Margulies, C. M. Mauck, S. A. Miller, Y. Wu, Y.-L. Wu, T. J. Marks, M. R. Wasielewski, Effects of crystal morphology on singlet exciton fission in diketopyrrolopyrrole thin films. *J. Phys. Chem. B* **120**, 1357–1366 (2016).

27. C. M. Mauck, P. E. Hartnett, E. A. Margulies, L. Ma, C. E. Miller, G. C. Schatz, T. J. Marks, M. R. Wasielewski, Singlet fission via an excimer-like intermediate in 3,6-Bis(thiophen-2-yl)diketopyrrolopyrrole derivatives. *J. Am. Chem. Soc.* **138**, 11749–11761 (2016).

28. Y. Dong, M. Taddei, S. Doria, L. Bussotti, J. Zhao, G. Mazzone, M. Di Donato, Torsion-induced nonradiative relaxation of the singlet excited state of meso-thienyl bodipy and charge separation, charge recombination-induced intersystem crossing in its compact electron donor/acceptor dyads. *J. Phys. Chem. B* **125**, 4779–4793 (2021).

29. A. B. Tamayo, M. Tantiwiwat, B. Walker, T.-Q. Nguyen, Design, synthesis, and self-assembly of oligothiophene derivatives with a diketopyrrolopyrrole core. *J. Phys. Chem. C* **112**, 15543–15552 (2008).

30. J. Zhou, H. Liu, S. Liu, P. Su, W. Wang, Z. Li, Z. Liu, Y. Chen, Y. Dong, X. Li, Singlet fission in colloidal nanoparticles of amphipathic diketopyrrolopyrrole derivatives: Probing the role of the charge transfer state. *J. Phys. Chem. B* **126**, 6483–6492 (2022).

31. K. Hanaoka, S. Iwaki, K. Yagi, T. Myochin, T. Ikeno, H. Ohno, E. Sasaki, T. Komatsu, T. Ueno, M. Uchigashima, T. Mikuni, K. Tainaka, S. Tahara, S. Takeuchi, T. Tahara, M. Uchiyama, T. Nagano, Y. Urano, General design strategy to precisely control the emission of fluorophores via a twisted intramolecular charge transfer (TICT) process. *J. Am. Chem. Soc.* **144**, 19778–19790 (2022).

32. M. Mahboub, H. Maghsoudiganjeh, A. M. Pham, Z. Huang, M. L. Tang, Triplet energy transfer from PbS(Se) nanocrystals to rubrene: The relationship between the upconversion quantum yield and size. *Adv. Funct. Mater.* **26**, 6091–6097 (2016).

33. S. Kumar, S. Kumar, P. Sharma, V. Sharma, S. C. Katyal, CdS nanofilms: Effect of film thickness on morphology and optical band gap. *J. Appl. Phys.* **112**, 123512 (2012).

34. R. Englman, J. Jortner, The energy gap law for radiationless transitions in large molecules. *Mol. Phys.* **18**, 145–164 (1970).

35. Z. Huang, Z. Xu, M. Mahboub, Z. Liang, P. Jaimes, P. Xia, K. R. Graham, M. L. Tang, T. Lian, Enhanced near-infrared-to-visible upconversion by synthetic control of PbS nanocrystal triplet photosensitizers. *J. Am. Chem. Soc.* **141**, 9769–9772 (2019).

36. T.-S. Ahn, R. O. Al-Kaysi, A. M. Müller, K. M. Wentz, C. J. Bardeen, Self-absorption correction for solid-state photoluminescence quantum yields obtained from integrating sphere measurements. *Rev. Sci. Instrum.* **78**, 086105 (2007).

37. A. Monguzzi, J. Mezyk, F. Scotognella, R. Tubino, F. Meinardi, Upconversion-induced fluorescence in multicomponent systems: Steady-state excitation power threshold. *Phys. Rev. B* **78**, 195112 (2008).

38. J.-K. Li, M.-Y. Zhang, L. Zeng, L. Huang, X.-Y. Wang, NIR-absorbing B,N-heteroarene as photosensitizer for high-performance NIR-to-blue triplet-triplet annihilation upconversion. *Angew. Chem. Int. Ed. Engl.* **62**, e202303093 (2023).

39. D. M. Cadena, J. K. Sowa, D. E. Cotton, C. D. Wight, C. L. Hoffman, H. R. Wagner, J. T. Boette, E. K. Raulerson, B. L. Iverson, P. J. Rossky, S. T. Roberts, Aggregation of charge acceptors on nanocrystal surfaces alters rates of photoinduced electron transfer. *J. Am. Chem. Soc.* **144**, 22676–22688 (2022).

40. Z. Xu, Z. Huang, C. Li, T. Huang, F. A. Evangelista, M. L. Tang, T. Lian, Tuning the quantum dot (QD)/mediator interface for optimal efficiency of QD-sensitized near-infrared-to-visible photon upconversion systems. *ACS Appl. Mater. Interfaces* **12**, 36558–36567 (2020).

41. J. R. Allardice, A. Thampi, S. Dowland, J. Xiao, V. Gray, Z. Zhang, P. Budden, A. J. Petty, II, N. J. L. K. Davis, N. C. Greenham, J. E. Anthony, A. Rao, Engineering molecular ligand shells on quantum dots for quantitative harvesting of triplet excitons generated by singlet fission. *J. Am. Chem. Soc.* **141**, 12907–12915 (2019).

42. J. T. DuBose, G. Szabó, J. Chakkamalayath, P. V. Kamat, Excited-state transient chemistry of rubrene: A whole story. *J. Phys. Chem. A* **126**, 7147–7158 (2022).

43. S. Kumazaki, Anti-stokes fluorescence of oxazine 1 in solution with continuous wave laser excitation at 785nm. *Chem. Phys.* **419**, 107–112 (2013).

44. M. Drobizhev, A. Karotki, M. Kruk, A. Krivokapic, H. L. Anderson, A. Rebane, Photon energy upconversion in porphyrins: One-photon hot-band absorption versus two-photon absorption. *Chem. Phys. Lett.* **370**, 690–699 (2003).

45. Q. Chen, C. Ye, S. Wan, S. Chen, Z. Liang, L. Li, Y. Shi, X. Wang, Multiwavelength hot-band excited triplet–triplet annihilation upconversion with Pt(ii)porphyrin complex/9,10-bis(phenylethynyl)anthracene and multiple encryption information applications. *J. Mater. Chem. C* **11**, 2069–2073 (2023).

46. A. V. Kachynski, A. N. Kuzmin, H. E. Pudavar, P. N. Prasad, Three-dimensional confocal thermal imaging using anti-Stokes luminescence. *Appl. Phys. Lett.* **87**, 023901 (2005).

47. H. Yu, Y. Chai, A. Tiemuer, Y. Han, H.-Y. Wang, Y. Liu, Frequency upconversion luminescence: New frontiers in bioapplications. *Coord. Chem. Rev.* **496**, 215379 (2023).

48. J. Saltiel, J. L. Charlton, W. B. Mueller, Nonvertical excitation transfer. Activation parameters for endothermic triplet energy transfer to the stilbenes. *J. Am. Chem. Soc.* **101**, 1347–1348 (1979).

49. A. Kalpattu, J. T. Fourkas, The mechanisms of endothermic triplet energy transfer in photochemical systems. *Chem. Phys. Rev.* **6**, 031305 (2025).

50. Z. Wang, M. Wu, X. Cui, F. Ge, P. Xiao, M. Li, H. Fu, Triplet–triplet annihilation upconversion with large anti-stokes shift. *ACS Nano* **19**, 25596–25616 (2025).

51. Y. Zhou, F. N. Castellano, T. W. Schmidt, K. Hanson, On the quantum yield of photon upconversion via triplet–triplet annihilation. *ACS Energy Lett.* **5**, 2322–2326 (2020).

52. Z. Huang, Z. Xu, M. Mahboub, X. Li, J. W. Taylor, W. H. Harman, T. Lian, M. L. Tang, PbS/CdS core–shell quantum dots suppress charge transfer and enhance triplet transfer. *Angew. Chem. Int. Ed. Engl.* **56**, 16583–16587 (2017).

53. R. Lai, Y. Liu, X. Luo, L. Chen, Y. Han, M. Lv, G. Liang, J. Chen, C. Zhang, D. Di, G. D. Scholes, F. N. Castellano, K. Wu, Shallow distance-dependent triplet energy migration mediated by endothermic charge-transfer. *Nat. Commun.* **12**, 1532 (2021).

54. W. Wu, H. Guo, W. Wu, S. Ji, J. Zhao, Organic triplet sensitizer library derived from a single chromophore (BODIPY) with long-lived triplet excited state for triplet–triplet annihilation based upconversion. *J. Org. Chem.* **76**, 7056–7064 (2011).

55. I. Moreels, K. Lambert, D. Smeets, D. De Muynck, T. Nollet, J. C. Martins, F. Vanhaecke, A. Vantomme, C. Delerue, G. Allan, Z. Hens, Size-dependent optical properties of colloidal PbS quantum dots. *ACS Nano* **3**, 3023–3030 (2009).

56. L. Huang, W. Wu, Y. Li, K. Huang, L. Zeng, W. Lin, G. Han, Highly effective near-infrared activating triplet–triplet annihilation upconversion for photoredox catalysis. *J. Am. Chem. Soc.* **142**, 18460–18470 (2020).

57. Y. Sasaki, M. Oshikawa, P. Bharmoria, H. Kouno, A. Hayashi-Takagi, M. Sato, I. Ajioka, N. Yanai, N. Kimizuka, Near-infrared optogenetic genome engineering based on photon-upconversion hydrogels. *Angew. Chem. Int. Ed. Engl.* **58**, 17827–17833 (2019).

58. J. Duan, Y. Liu, Y. Zhang, Z. Chen, X. Xu, L. Ye, Z. Wang, Y. Yang, D. Zhang, H. Zhu, Efficient solid-state infrared-to-visible photon upconversion on atomically thin monolayer semiconductors. *Sci. Adv.* **8**, eabq4935 (2022).

59. T. N. Singh-Rachford, A. Nayak, M. L. Muro-Small, S. Goeb, M. J. Therien, F. N. Castellano, Supermolecular-chromophore-sensitized near-infrared-to-visible photon upconversion. *J. Am. Chem. Soc.* **132**, 14203–14211 (2010).

60. Y. Niihori, Y. Wada, M. Mitsui, Single platinum atom doping to silver clusters enables near-infrared-to-blue photon upconversion. *Angew. Chem. Int. Ed. Engl.* **60**, 2822–2827 (2021).

61. M. Mahboub, Z. Huang, M. L. Tang, Efficient infrared-to-visible upconversion with subsolar irradiance. *Nano Lett.* **16**, 7169–7175 (2016).

62. W. Liang, C. Nie, J. Du, Y. Han, G. Zhao, F. Yang, G. Liang, K. Wu, Near-infrared photon upconversion and solar synthesis using lead-free nanocrystals. *Nat. Photonics* **17**, 346–353 (2023).

63. R. Sun, J. Zang, R. Lai, W. Yang, B. Ji, Near-infrared-to-visible photon upconversion with efficiency exceeding 21% sensitized by inas quantum dots. *J. Am. Chem. Soc.* **146**, 17618–17623 (2024).

64. S. Izawa, M. Hiramoto, Efficient solid-state photon upconversion enabled by triplet formation at an organic semiconductor interface. *Nat. Photonics* **15**, 895–900 (2021).

65. N. Tripathi, M. Ando, T. Akai, K. Kamada, Efficient NIR-to-visible upconversion of surface-modified PbS quantum dots for photovoltaic devices. *ACS Appl. Nano Mater.* **4**, 9680–9688 (2021).

66. N. Kiseleva, P. Nazari, C. Dee, D. Busko, B. S. Richards, M. Seitz, I. A. Howard, A. Turshatov, Lanthanide sensitizers for large anti-stokes shift near-infrared-to-visible triplet–triplet annihilation photon upconversion. *J. Phys. Chem. Lett.* **11**, 2477–2481 (2020).

67. Z. Huang, X. Li, M. Mahboub, K. M. Hanson, V. M. Nichols, H. Le, M. L. Tang, C. J. Bardeen, Hybrid molecule–nanocrystal photon upconversion across the visible and near-infrared. *Nano Lett.* **15**, 5552–5557 (2015).
